# Modelling individual and cross-cultural variation in the mapping of emotions to speech prosody

**DOI:** 10.1038/s41562-022-01505-5

**Published:** 2023-01-16

**Authors:** Pol van Rijn, Pauline Larrouy-Maestri

**Affiliations:** 1 Max Planck Institute for Empirical Aesthetics, Frankfurt am Main, Germany; 2 Max Planck–NYU Center for Language, Music, and Emotion, New York, NY USA

**Keywords:** Human behaviour, Science, technology and society

## Abstract

The existence of a mapping between emotions and speech prosody is commonly assumed. We propose a Bayesian modelling framework to analyse this mapping. Our models are fitted to a large collection of intended emotional prosody, yielding more than 3,000 minutes of recordings. Our descriptive study reveals that the mapping within corpora is relatively constant, whereas the mapping varies across corpora. To account for this heterogeneity, we fit a series of increasingly complex models. Model comparison reveals that models taking into account mapping differences across countries, languages, sexes and individuals outperform models that only assume a global mapping. Further analysis shows that differences across individuals, cultures and sexes contribute more to the model prediction than a shared global mapping. Our models, which can be explored in an online interactive visualization, offer a description of the mapping between acoustic features and emotions in prosody.

## Abstract (web summary)

A mapping between emotions and speech prosody is commonly assumed. This study shows, using Bayesian modelling, that differences across individuals, cultures and sexes contribute more to the model prediction than a shared global mapping.

## Main

Early studies in emotion science focused on showing similarities of emotions across cultures^1^. More recently, renewed efforts have been made by estimating variability in emotional language^2^, facial expressions^3^, physiological measurements^4^ and non-verbal vocalizations^5^ across individuals and cultural groups. Here we build on this new wave of research by estimating and examining sources of variability in emotional prosody at scale.

There are three influential families of emotion theories that predict different degrees of variability: affect program, psychological constructivist^6^ and appraisal theories^7^. Affect program theories, including the influential basic emotion theory^8^, assume the existence of neural signatures for specific emotions. While the framework accommodates variability (such as the in-group effect^9^ predicting that emotions are better understood by a member of the same community^10^), these theories seldom predict systematic sources of variability in emotion expression and recognition. Constructionist theories, in contrast, which deny the existence of any hard-wired links dedicated to specific emotions^11^, predict that emotion should vary widely across situations, individuals and cultural groups. Finally, variability is inherently predicted by appraisal theories^7^, which assume that each emotion is caused by its appraisal pattern^12^. Small changes in the appraisal pattern may lead to a different action tendency—a tendency to flee might become a tendency to fight. The exact appraisal pattern depends on the internal state of the listener and thus predicts variability.

**Fig. 1:**
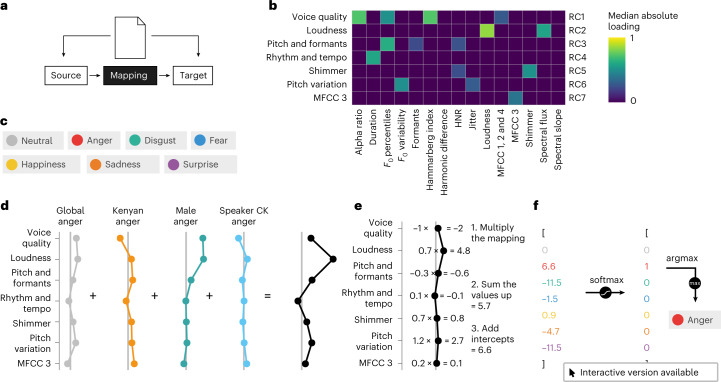
Conceptualizing the relationship between acoustic features and emotional speech as a mapping problem. **a**, Emotion recognition is conceptualized as a mapping problem. The mapping describes how the source (acoustic features) can be related to the target (intended emotions). **b**, Factor analysis reveals seven acoustic dimensions that relate to perceptual qualities of speech prosody. To ease the visualization of the data, weak loadings (<0.45) are not shown in the loading plot. The full loading plot can be found in Supplementary Fig. 4. MFCC, mel-frequency cepstral coefficient; HNR, harmonics-to-noise ratio. **c**, The six basic emotions and ‘neutral’ are used as mapping targets. **d**, The model learns a multilevel mapping, consisting of a mapping that exists in all corpora as well as mapping deviations on the basis of certain grouping variables, such as culture or speaker. In this particular example, the mapping for ‘anger’ for a male Kenyan English speaker (speaker CK) is depicted. **e**, To obtain a prediction for a specific emotion, we take the mapping (**d**) and multiply it by the respective acoustic factor values of some input stimulus, sum the values and add the intercepts. **f**, Predictions for all six emotions (as in **e**). ‘Neutral’ always obtains the prediction 0, as it is the pivot category. The seven values are converted into probabilities (softmax), and the emotion category with the highest probability is the category prediction for some input stimulus. For an interactive version of **d**–**f**, see http://mapping-emotions.pol.works.

In the present study, we describe the mapping between speech prosody and emotion by using Bayesian multilevel multinomial logistic regression models (Fig. 1a). Speech prosody is characterized by variations in pitch, loudness, timing and voice quality (Supplementary Discussion 2). Here we use a common feature set^13^ that spans most prosodic dimensions^14–16^. To obtain interpretable regression coefficients, we reduced the dimensionality to seven uncorrelated acoustic factors (Fig. 1b). Additional analyses described in Supplementary Methods 3 show that the factor solution is relatively robust across the most common languages and countries.

We collected an array of emotional speech recordings by adopting standards^17^ to query, filter and annotate the possible datasets (Supplementary Methods 1). For a corpus to be included, emotion annotations must be present, and the corpus must contain recordings of sentences (that is, no syllables, non-verbal vocalizations or single-word sentences). In the analyses presented in this manuscript, we include corpora that only contain healthy adult speakers, for which the intended expression is known and that we were granted access to (see Supplementary Discussion 1, Supplementary Table 1 and Supplementary Methods 2 for more details). While some researchers explore extended sets of emotion categories^18–20^, the majority of emotion research has centred on the limited set of basic emotions. We therefore focused on these emotions (Fig. 1c). The full list of corpora accompanies the release of this publication, and new corpora can be proposed via an online form and will be published upon review: emotional.speechcorpora.com.

The mapping between acoustic features and intended emotional speech can be studied either by modelling the relationship between acoustic features and emotional expression^21^ (studying production), as we do, or by analysing human recognition rates (perception)^17^. The second approach mostly relies on meta-studies; however, this approach is fundamentally limited since it relies on effect sizes and standard errors, discarding relevant information about individual samples and differences within the tested population. We overcame this limitation by using Bayesian inference models to estimate the mapping at different levels—enabling the quantification of cultural, speaker and sex differences. We pursued this goal by studying a collection of intended emotional prosody productions, including 432 individuals from around the world, speaking 2,963 different sentences. Altogether, this represents 3,252 minutes of intended emotional speech. This collection of emotional prosody together with Bayesian inference models allows us to study the mapping at scale and provide answers to the following questions: how variable is the mapping within and across datasets, and what effect do moderators (such as speakers or cultures) have on the mapping?

When studying the relationship between acoustic features and intended emotions, one can study four aspects: reliability, specificity, generalizability and validity of the mapping^22^. High reliability means that the same emotion is expressed by a common set of features. Our first research question addresses the reliability of the mapping within and across corpora of speech prosody. Specificity means that a pattern of acoustic features refers to one and only one emotion. In other words, high specificity implies a good classification performance. By contrasting models allowing for different sources of variability, we address a concept similar to specificity. Generalizability means that differences across different populations have sufficiently been accounted for. In our final analysis, we identified which levels of analysis—for example, cultural, individual or sex differences—have the largest contribution to the model prediction. High validity signals that the person expressing the utterance is actually in the expected emotional state. However, as we elaborate in Supplementary Discussion 3, estimating validity is not so straightforward. Consequently, in this Article, we evaluate the reliability, specificity and generalizability of the mapping from basic emotions to speech prosody in productions.

To provide answers to our research questions, we used Bayesian multilevel multinomial logistic regression models. Internally, the model computes a linear predictor for each emotion. The emotion with the highest value is the emotion predicted by the model. Each predictor consists of an intercept—accounting for possible imbalances in the base rate of emotion labels—and a series of coefficients for each of the seven acoustic features describing the mapping between speech prosody and emotions. In addition to this ‘global mapping’, we compute a deviation for different levels of analysis. One challenge in modelling this deviation is that for some groups there are fewer data points (for example, there are more Indian than Dutch samples), which would make the estimates for the small groups less reliable. A solution to this problem is partial pooling, which adjusts estimates for groups with small sample sizes or with extreme values more towards the grand mean of the data. This mechanism—often referred to as shrinkage—makes the predictions more realistic and the model less likely to overfit^23^.

Here we are primarily interested in the acoustic coefficients, as they are estimates of the mapping. This yields a multilevel mapping of acoustic factors (Fig. 1d). To obtain a prediction for a specific emotion, we multiply the multilevel mapping by an input sample that we want to obtain a prediction for. The values of the multiplication are added together along with the intercepts (Fig. 1e). This is performed for all six emotions. ‘Neutral’ always obtains the prediction 0, because it is the pivot category. The predictions for the six emotions and ‘neutral’ are converted to probabilities, and the model selects the emotion with the largest probability (Fig. 1f). For all models reported in the paper, we provide an online, interactive version of the model similar to Fig. 1d–f, which enables the visualization of model predictions for existing samples or obtaining insights into what the model has learned. All interactive models can be found at http://mapping-emotions.pol.works.

Our model design overcomes several pitfalls of traditional meta-analysis. First, it estimates mapping differences at granular levels of analysis—for example, on a speaker level. It also avoids false confidence based on removed variation by averaging, and it accounts for imbalances in sampling (such as different numbers of stimuli per culture). Finally, since all recordings are processed with the same pipeline, the extracted features are computed identically and are thus comparable across corpora, which is not necessarily the case for meta-studies^24^.

## Results

### Overview

Guided by our modelling framework, our data analysis proceeded as follows. First, to describe the reliability of the mapping, we examined the variability in the mapping estimates within and across different corpora. Then, to address the specificity of the mapping, we performed a contrastive model comparison exploring which model best fits the data, while punishing overly complex models. Finally, we uncovered which levels of analysis contribute the most to the prediction of the model and supported the findings with a correlation and variability analysis.

### Verifying the Bayesian inference models

Prior to the main analysis, we showed that our Bayesian multinomial logistic regression models perform equally well in the classification task as do support vector machines (SVMs), which have been extensively used in emotion classification from audio^25^ (see the Methods for the hyperparameters used). Emotion classification performance is often expressed as unweighted average recall (UAR)^26^, which is the average recall across all emotion categories while accounting for slight imbalances in the base rate of the categories. Using fourfold leave-speaker-out cross-validation, we showed that the SVM obtains a similarly high UAR score as the Bayesian regression model (25.5% and 22.7% UAR, respectively; Bayesian estimation of the mean paired difference, −4%; 89% credible interval, −12% to 4%), indicating that the Bayesian multinomial logistic regression performs comparably to a common baseline. Here we evaluated model prediction; however, in the main analysis we use the Bayesian logistic regressions as inferential models. Thus, the objective is not to optimize model prediction for unseen data but rather to explore what the models have learned.

### High reliability within corpora and poor reliability across corpora

**Fig. 2:**
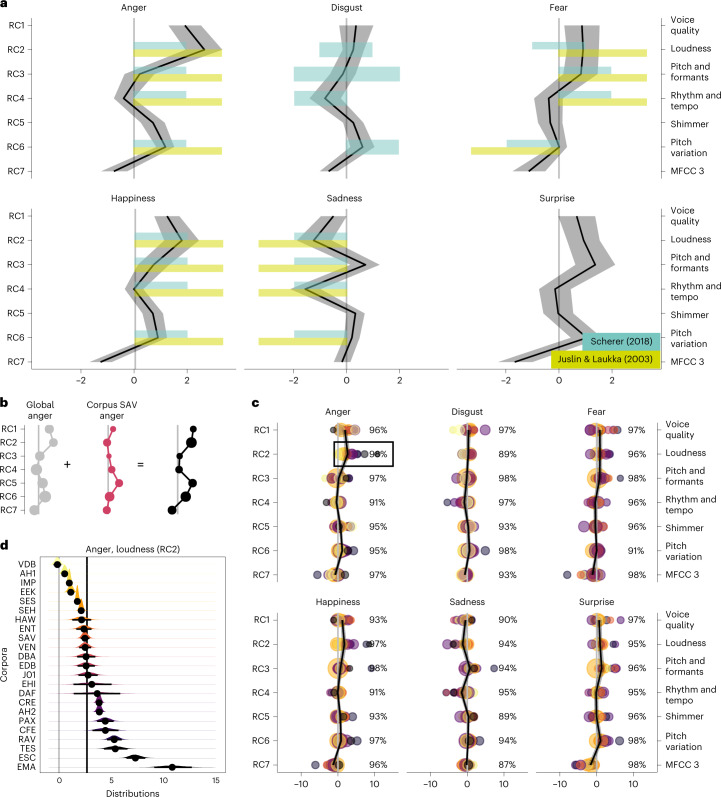
Variability across datasets as shown by model coefficients for each acoustic factor across all corpora (population-level effect) and deviations per corpus (group-level effect). **a**, The model estimates a coefficient for each of the seven acoustic factors (RCs) and a group-level deviation per corpus. The black line is the average coefficient across corpora, and the grey area around the line is an 89% credible interval. To put our model estimates in some context, we include the empirical findings from two reviews on acoustic profiles of emotions^16,27^. Juslin and Laukka^16^ only distinguish between positive and negative; Scherer^27^ distinguishes between a little and very negative or positive. **b**, The model internally combines the population- and group-level effects. In this particular example, the estimates for ‘anger’ in the corpus ‘SAV’ for RC1–7 are depicted. The black line is the combined mapping, which is plotted in the following subplots. The larger the size of the dots, the smaller the credible interval. **c**, Each coloured dot represents a combined estimate for a specific corpus (average across corpora + corpus-specific estimate) of an acoustic factor (RC1–7) for all emotions. Large dots indicate small credible intervals (that is, narrow distributions). The black line is the average coefficient, and the area around the line is an 89% credible interval. The vertical grey line indicates 0. The percentage on the right of each subplot is the *I*^2^ value. **d**, Zoomed-in version of factor RC2, ‘loudness’. The combined estimates per corpus (*n* = 4,000) rarely overlap. The black line below the distribution indicates an 89% credible interval. The vertical black line is the average coefficient (population-level effect), and the grey line is positioned at the origin.

We next fit a model that estimates a coefficient for each of the seven acoustic factors across the six emotions (Fig. 2a). On top of this ‘global mapping’, we computed a corpus-specific deviation from this coefficient (Fig. 2b). In doing so, we measured the variability of the mapping within a corpus and across corpora. The estimates are depicted in Fig. 2c. The variability within a corpus is characterized by the spread of the distribution of estimates. Wide distributions indicate more variability for the given estimate in a corpus (smaller dots indicate greater variability in Fig. 2b,c). Variability across corpora can be described by the overlap in the estimated distributions across corpora. If there is a poor overlap of the distributions, then there is a great deal of variability across corpora.

While the estimated emotion coefficients across corpora mostly match with empirical predictions from two reviews on emotion-specific acoustic profiles^16,27^ (Fig. 2a), there are some disagreements—for example, happiness is predicted to have a higher speech rate and sadness to have a lower pitch. Such differences are to be expected because the factor scores do not relate one-to-one to the raw acoustic features, and there is a large spread in the coefficients estimated for the different corpora (Fig. 2c). This variability across corpora is even more striking, as shrinkage in multilevel models pulls observations from small corpora or extreme observations closer to the grand mean.

In Fig. 2d, we zoom in on a single factor (RC2, loudness, for anger) and can see that the estimates for the coefficients are rather tight (that is, the distribution of estimates is narrow). This implies that the mapping of a certain acoustic factor to an emotion label is consistent within a corpus. However, across corpora, we can observe that the credible intervals of the distributions are only partially overlapping, which means that the estimates from one corpus to another often differ. If the mapping between acoustic features and emotion labels were identical across corpora, we would expect a greater degree of overlap. Note that high variability does not imply low emotion recognition but is merely a justification to use moderators in the analysis. Given the observed variability in the estimates across corpora, the next step is to investigate the origin of the variability.

The objective here is to show the convergence of evidence (or the lack thereof) across studies. In meta-studies, each study is treated as an individual sample with its effect size and standard error. Some degree of variation across studies is expected due to minor sampling differences in the population, which should be smaller for larger sample sizes. Measuring the amount of heterogeneity among studies is key to the question of convergence, as large variability might indicate that studies measure distinct concepts, or moderators need to be included. We borrow the *I*^2^ metric from meta-analysis, which describes the proportion of total variation in study estimates due to heterogeneity^28^ (see the Methods for the details). Here we compute *I*^2^ separately for each factor and emotion and treat the estimates from single corpora as separate studies. The *I*^2^ values are shown on the right of each subplot in Fig. 2c. The analysis confirms that there is a great deal of variability in estimates across corpora and that this variance is larger than what would be expected on the basis of sampling variance alone.

### Models only assuming a global mapping are outperformed

Given that estimates across corpora are heterogeneous, we ran a series of models accounting for different moderators. Every model estimates a separate intercept for each corpus to account for possible imbalances in the base rate of emotions across corpora. Models are compared to each other using the widely applicable information criterion (WAIC), which provides an approximation of the out-of-sample deviance while penalizing overly complex models, which tend to overfit the data (Supplementary Methods 4). Thus, the relative WAIC difference between contrasting models is of importance, where lower WAIC values indicate a better model fit.

**Fig. 3:**
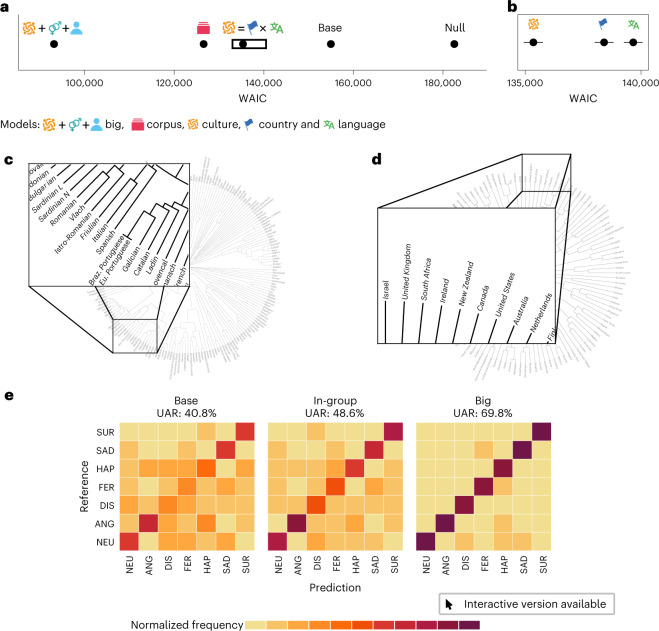
Model comparison and sensitivity. **a**, Model comparison using the WAIC. The models are arranged by their WAIC score, where lower WAIC values indicate a better model fit. The following models are shown, from right to left: the null model containing only intercepts; the base model estimating the global mapping; the in-group model estimating the interaction between country and language (we call this interaction ‘culture’); the corpus model from Fig. 2b; and the big model, which is the in-group model additionally modelling speaker and sex differences. The error bars are standard errors of the WAIC. **b**, Zoomed-in version of the black box in **a**, showing the WAIC of the in-group models modelling the group-level effect of countries, languages or the interaction of both. The icons are introduced in detail in Supplementary Methods 2. **c**, UPGMA-generated language tree from Beaufils and Tomin^29^. **d**, UPGMA-generated culture tree from Euclidian distances among the Hofstede^30^ dimensions. **e**, Confusion matrices predicting the dataset for the base, in-group and big models. Overall performance is expressed in UAR. Each cell contains a recall value. The recall values for each row are normalized and sum to 1. SUR, surprise; SAD, sadness; HAP, happiness; FER, fear; DIS, disgust; ANG, anger; NEU, neutral. All models in **a**,**b** can be explored using an interactive visualization; see http://mapping-emotions.pol.works.

As a lower boundary, we fit an intercept-only model estimating an intercept for each emotion and corpus. The ‘base’ model additionally estimates a coefficient for each acoustic factor. As shown in Fig. 3a, the base model is much better than the intercept-only model.

We then fit a series of models inspired by the emotion dialect theory^10^, on the basis of the ‘in-group’ effect. One way to model this membership is to add a group-level effect for languages and countries. As shown in Fig. 3b, the language model and the country model perform similarly well (the country model is slightly better). However, this initial approach was limited in that we treated languages and countries as discrete categories and ignored the proximity of different languages and countries to one another—for example, Dutch being linguistically closer to English than to Hindi. To model this proximity, we computed the Euclidean distances among languages and countries. Language distance is modelled as lexical distance^29^, and differences across countries are captured on the Hofstede cultural dimensions^30^. As depicted in Fig. 3c,d, the language and country trees reconstructed from the distances^31^ contain meaningful associations. For example, in the language tree, Brazilian Portuguese is closer to European Portuguese than it is to Spanish, and Romance languages are grouped together; for the country model, the Anglo-Saxon countries (the United States, Canada, Australia and New Zealand) are grouped together. However, models incorporating this complex hierarchical relationship did not converge. As a pragmatic solution, we therefore modelled ‘culture’ as the combination of the categories ‘language’ and ‘country’, as this enables useful distinctions (such as between American and Canadian English). As depicted in Fig. 3b, this model is better than the language or country model.

As shown in Fig. 3a, the culture model is outperformed by the corpus model from the reliability analysis (see the lower, non-overlapping WAIC value for the corpus model), as the grouping variable ‘corpus’ contains the same grouping information as in ‘culture’—each corpus is usually assigned to one country and one language—and additionally consists of more specific information potentially relevant for the communication of emotion. For example, speakers are often recruited from the same area or institution (for example, the same city or university), targeting a more specific social group^9^. However, the grouping variable ‘corpus’ is—in contrast to ‘language’ or ‘country’—an artificial construct that is transcended by a series of more realistic constructs, such as cultural proximity and social belonging. We therefore extend the culture in-group model (and not the corpus model) by adding sex and individual speaker differences. As shown in Fig. 3a, this ‘big’ model outperforms all other models.

The confusion matrices in Fig. 3e reveal that with increasing model complexity, the misclassifications by the model are reduced (darker diagonals), and hence the overall UAR per model increases (40.8% for base, 48.6% for the best in-group model and 69.8% for the final model). For example, in the base model, ‘happiness’ is often misclassified as ‘anger’ and ‘neutral’ as ‘sad’. In contrast to the WAIC, confusion matrices do not penalize overfitting models. And one would expect that with increasing model complexity, models will better fit (or even overfit) the data. However, group-level effects can have a regularizing effect due to shrinkage and hence reduce the risk of overfitting. The confusion matrices show that the models capture the trend in the data and are better at it with increasing model complexity.

### Relevance of culture, sex and individual differences

**Fig. 4:**
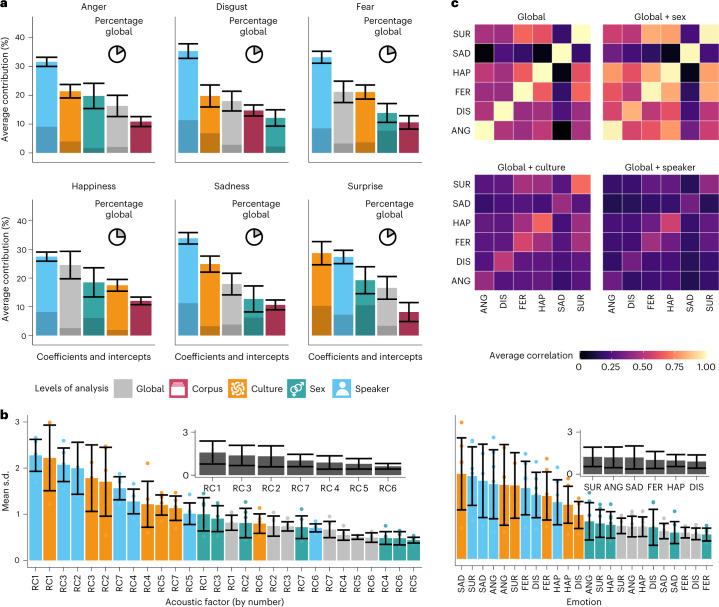
Differences in the mapping across cultures, sexes and individuals. **a**, Contributions of different levels of analysis to the model prediction. Each panel shows the mean contributions of different levels of analysis in all cases in which the emotion was predicted. The error bars are standard deviations across single posterior draws (*n* = 4,000). The colour of each bar indicates the level of analysis. The darker section of each bar represents the contribution of the intercept. The lighter section represents the contribution of the acoustic coefficients. The pie chart in the upper right of each panel is the contribution of the global mapping to the full prediction. **b**, Variability in the coefficients for different levels of analysis. For each group level, emotion and acoustic factor, a standard deviation was computed on all coefficients. In both panels, the average standard deviation is plotted by the acoustic factor (left, *n* = 6) and the intended emotion (right, *n* = 7). The error bars are in standard deviations. The subplots collapse over the different levels of analysis. **c**, Correlation across mappings. The upper left panel shows the mappings of all emotions correlated with each other. The diagonals are always 1. The remaining three panels show correlations between the global mapping and sex, cultural or speaker difference. The fill colour is the average correlation (Pearson).

To examine how individual levels of the mapping contribute to the prediction of the model, we computed the contribution of each level of analysis to the prediction of the model. We first obtained the model prediction on the data that the model was fitted on (as in Fig. 3e), and we then measured how much each group level contributes to the value for the predicted emotion (Fig. 4a). In all emotions (except ‘surprise’), individual differences have the greatest impact on the model prediction. The second most important level of analysis is culture for most emotions, followed by the global mapping or sex differences. Remarkably, only 20–25% of the model prediction originates from the global mapping, as depicted by the pie charts in the upper right corner of each panel in Fig. 4a.

As depicted in Fig. 4a, the intercepts (marked by the darker colours) play a subordinate role in the prediction of the emotion. In addition, the intercept of the corpus has the smallest contribution to the final prediction in all emotions except for ‘disgust’.

### Variability in coefficients is the largest for speakers and cultures

While in the previous analysis the contributions of different levels of analysis were estimated in the original data, the current variability analysis was performed on the model estimates regardless of the data. We extracted the posterior estimates for each acoustic factor, each emotion and each group level and computed the average standard deviation as a metric of the variability of the estimates. As depicted in Fig. 4b, most variability can be found in the ‘speaker’ and ‘culture’ estimates. Overall, the first three acoustic factors (voice quality, loudness, and pitch and formants) show the most variability (see the subplot in the left panel of Fig. 4b). The remaining factors (except RC7, MFCC 3) have decreased variability corresponding to their component numbers. The variability results per emotion also show that the estimates for ‘speaker’ and ‘culture’ are the most variable. All estimates for the emotions are variable, although ‘surprise’, ‘anger’ and ‘sadness’ appear to be slightly more variable than the other three emotions (see the subplot in the right panel of Fig. 4b).

### Confusion between the production of emotions across cultures, sexes and individuals

In the next correlation analysis, we again used the coefficient estimates. We started by correlating the global mapping across emotions. As depicted in the upper left panel of Fig. 4c, ‘sadness’ is the only emotion with a distinct profile, as it has only a strong correlation with itself and low correlations with all other emotions. Interestingly, the profiles of the other emotions correlate more strongly with each other, especially the correlations among the profiles for ‘fear’, ‘happiness’ and ‘surprise’.

In three further analyses, we described the relationship between emotions across sexes, cultures and individuals. A first analysis showed that the mapping for a specific emotion correlates the most strongly with the mapping for the same emotion of the other sex (right panel of Fig. 4c). For instance, female anger is, on average, closer to male anger than to any other emotion. When compared with the global mapping, adding sex further increases the correlation among the profiles of ‘fear’, ‘happiness’ and ‘surprise’.

The addition of ‘culture’ or ‘speaker’ to the global mapping leads to a strong decrease in the overall correlations across emotions, indicating that the mapping for individual cultures and speakers is relatively distinct. The overall drop in correlation is greater for speakers than for cultures, confirming the pattern of results in the previous analyses (Fig. 4a). Nonetheless, the diagonals are mildly preserved, indicating that the mapping for a given emotion is more similar across speakers and cultures than to another emotion.

## Discussion

Studies have shown that there is substantial variability in the mapping of emotions to facial expressions^3^, physiological measurements^4^ and non-verbal vocalizations^5,32^ indicating that expressions of emotions vary widely between contexts and cultures. In the present study, we investigated the relationship between speech prosody and emotions by modelling the relation as a mapping problem.

Using a Bayesian modelling framework, we examined the mapping between acoustic features and emotions in speech recordings at multiple levels of analysis. Our focus was to describe the mapping by investigating three requirements to assume the existence of a mapping: reliability, specificity and generalizability.

Guided by this conceptual framework, we collected a set of intended emotional speech samples. To encourage future research to use larger and more diverse emotional speech corpora, we made available a continuously updated list of corpora of emotional prosody, including access information and rich annotations, which simplifies the process of preprocessing and obtaining access to the corpora.

Concerning the reliability of the mapping, we showed that the mapping within a corpus is relatively reliable, whereas the mapping across corpora is highly variable. The large variability across corpora implies that findings from single corpora do not necessarily transfer to other corpora of emotional prosody and thus that results from single corpora need to be taken with caution. The low reliability across corpora fits well with the large amount of disagreement in the reported acoustic profiles for a single emotion. For example, ‘sadness’ has been associated with a low^33^, moderate^34^ and increased standard deviation of fundamental frequency^14^.

Here we showed that models computing a multilevel mapping based on the corpus instead of the culture yield better results. We argue that the grouping variable ‘corpus’ is unlikely to be a concept relevant for the communication of emotion but instead is transcended by a series of more plausible concepts, such as cultural proximity and social belonging.

We also examined the specificity of the mapping. As indicated by the initial verification analysis, the Bayesian regression models obtain 22.7% UAR (fourfold cross-validation), which is above the 14.3% chance level and shows that at least a part of the mapping is shared. This is also supported by the analysis shown in Fig. 4a indicating that the global mapping contributes ~20–25% to the final prediction of the model. However, with a series of increasingly complex models, we showed that models accounting for individual, cultural and sex differences outperform models assuming only a global mapping, indicating that there are many cultural and individual differences in the mapping.

Lastly, we examined the generalization of the mapping between emotions and speech prosody. We showed that the model predictions are mainly driven by individual and cultural differences, which fits our finding that most variability is found in estimates for cultures and speakers, and correlations for the mappings between individual cultures and speakers are generally low.

It is important to note that the pattern of results observed in this investigation was potentially influenced by the fact that we studied recordings in which the intended emotion was known. These kinds of recordings often include acted databases. A body of research indicates that there are differences between acted and spontaneous utterances of emotional prosody^35–37^. One key concern when working with acted material is that the produced emotional stimuli are stereotypical and thus are not necessarily expressions of emotion used in daily life^4,22^. Given this consideration, one might hypothesize that there should be a large overlap in the mapping across corpora, as stereotypes may be culturally shared. However, our results show that global mapping contributed roughly a quarter of the model prediction. Furthermore, the boundary between spontaneous and acted corpora might not be so clear, as both types of corpora heavily rely on actors, as we argue in Supplementary Discussion 1.

We note that the selection of seven acoustic factors is not entirely justifiable—a larger number of factors could also be plausible. Ideally, one would like to contrast models that rely on different acoustic representations; but when another feature is added to the model, the number of parameters that the model would need to estimate substantially increases. We therefore used a reduced set of factors that load on perceptually meaningful dimensions^7,14^ (Fig. 1b), which makes it easier to interpret model predictions and learned parameters. Furthermore, the models developed in this paper are only rough approximations of the mapping between intended emotions and speech prosody productions. Preferably, one would use more group-level effects and moderators of a higher quality. However, by adding extra group levels—especially if there are many levels (for example, 2,963 different sentences)—one easily hits the limits of computational tractability. In addition, more precise moderators are often not available and are not reconstructable a posteriori. For example, the country the corpus was recorded in does not necessarily reflect the country the speaker was born in and is likely to be less informative than the country of birth or even finer cultural subgroups, but such information is often not available. Given these considerations, we constructed the models with the best moderators available, which are theoretically motivated and of sufficient quality. Granting these limitations and caveats, the methods developed here could be fruitfully applied to any other mapping problem, such as the mapping of emotions to non-verbal vocalizations. Moreover, a reparameterization of the model (for example, replacing the multinomial logistic regression with a plain logistic regression) can drastically bring down the model complexity.

In the present investigation, we have shown that there is considerable variability in the mapping between emotions and speech prosody and that the global mapping contributes roughly a quarter to the model predictions. The observed variability is compatible with all three theories of emotion. Constructivist theories predict that emotions are perceptually variable instances interpreted by a perceiver that are grouped together by their function or purpose rather than by similar features^38^. Appraisal theories predict that the same stimulus might lead to different appraisal patterns. Affect program theories have historically been interested in finding similarities in how emotions are produced across cultures; however, the notion of emotion families^8^ is an in-theory explanation for large variability. Emotion families imply that occurrences of the same emotion might refer to different granularities of the same emotion (for example, ‘hot anger’ as a subtype of ‘anger’). This problem becomes apparent when meta-studies summarize over emotion labels. For example, Juslin and Laukka^16^ count the emotions ‘afraid’, ‘anxiety’, ‘frightened’, ‘scared’, ‘panic’, ‘terror’ and ‘worry’ all to ‘fear’, but it is disputable whether these all refer to the same concept. This problem is further amplified once emotional concepts are translated. Unfortunately, this issue is often neglected. For instance, Cowen et al.^39^ merely rely on the translation of the emotion categories by a single co-author. Recent studies comparing word meanings across many languages found emotional terms to be highly culture-dependent compared with object terms such as ‘mountain’^2,40^. This might have contributed to the overall low correlation found across cultures. This poses a problem of construct validity when doing cross-cultural research^41^. In the present study, we considered the emotion to be identical only if the English translation given by the author of the corpus is identical—for example, we considered ‘fear’ and ‘anxiety’ to be different emotions. While this pragmatic approach clearly has its limitations, the correlation analysis presented in Fig. 4c shows that the correlation between mappings across cultures is the highest for the same emotion compared with other emotions. This indicates that the emotion labels in the corpora refer to closely related or identical concepts. Our findings are thus compatible with all three families of emotion theories.

Emotion theories are often discussed in light of findings of high variability and low specificity^3,4,22^. However, the differences in predicted outcomes between the three theories are at most those of emphasis rather than of opposition. This makes it hard to specify how much evidence of variation or of specificity would be needed to support each view. Meta-analytic investigations cannot directly tackle these questions. This discussion also highlights another core problem in emotion science^42^: emotion theories often make vague predictions, and the line of argumentation is frequently indirect. For example, given the previously introduced concept of ‘refinement’, it is unclear how much variability one would predict to measure distinct acoustic patterns across languages attributed to differences caused by the translation. A more efficient method to address these key questions would be to experimentally address them^43^. This has been made possible by the development of modern algorithms that allow sampling from human prototypes^44^ and rapid improvements in speech synthesis^45^.

In this manuscript, we explored the mapping between acoustic features and emotions in a large sample of intended emotional speech recordings. Not only are our findings of individual, cultural and sex differences compatible with results from other modalities^3,4,22^, but we also quantify them in the domain of speech prosody.

## Methods

### Corpora

For a comprehensive overview of available corpora of emotional prosody, we used three search strategies querying literature databases and data repositories as well as scanning existing review papers. The corpus candidates were hand-filtered using a predefined annotation scheme. We requested access to 200 corpora but obtained access to only 42. In total, 24 corpora passed our requirements and were included in the analysis^18,19,34,46–66^. See Supplementary Table 2 for more information. The full list of corpora has been released in conjunction with this publication and will be continuously updated as new corpora are published: emotional.speechcorpora.com. For each of the remaining 24 corpora, we made sure that the following annotations are present: speaker, sex, country, language, emotion intensity, emotion induction procedure, recording modality, normal or pseudo-speech, number of repetitions, speaker type, corpus, whether the corpus was fully crossed, the year the corpus was published in, and whether the corpus was validated or not. See Supplementary Materials 2 for a description of each of the annotations.

### Preprocessing

To identically process all the corpora, we ran the following preprocessing steps. First, we made sure that there were no sounds other than speech that could disturb the acoustic feature extraction, such as background music. For one corpus^63^, we had to segment the speech from longer fragments into sentences. This was done with an adaptive algorithm changing a loudness threshold and a minimal silence duration in Praat^67^ using Parselmouth^68^. If there were only video recordings of the spoken sentence, audio was extracted from the video signal. Finally, all recordings were converted to mono and downsampled to 16,000 Hz. For each file, we encoded the following information into the filename: corpus, intended emotion, sentence code, speaker, repetition and emotional intensity (if this was explicitly requested by the experimenter).

### Acoustic analysis

Here we use the eGeMAPS standard feature set^13^, as it has been extensively used for the classification of emotion. While other performative handcrafted^69^ or learned^70^ feature representations are available, they are less applicable to factor analysis due to their dimensionality. A description of the features contained in eGeMAPS can be found in Supplementary Table 2.

### Factor analysis

Of the 88 features, 74 are correlated at least 0.3 with at least one other feature, suggesting reasonable factorability. The Kaiser–Meyer–Olkin measure of sampling adequacy is 0.87, and Bartlett’s test of sphericity is significant (*χ*^2^(3,828) = 9,429,598, *P* < 0.01). Principal components analysis with Varimax (orthogonal) rotation was conducted using the R package psych^71^ because the primary purpose was to reduce the dimensionality of the features while reducing their correlation.

We selected a seven-factor solution (see Supplementary Methods 3 for a justification). The factors explain 12%, 11%, 10%, 10%, 6%, 4% and 4% of the variance (57% in total). Factor 1, ‘voice quality’, mainly loads on alpha ratio, Hammarberg index, and MFCC 1, 2 and 4 (see Supplementary Fig. 4d for the loading plot). Factor 2, ‘loudness’, loads mainly on loudness and spectral flux. Factor 3, ‘pitch and formants’, loads on fundamental frequency, on the formants (*F*_1–3_) and mildly on HNR. Factor 4, ‘rhythm and tempo’, mainly loads on durational features. Factor 5, ‘shimmer’, loads on shimmer and mildly on HNR. Factor 6, ‘pitch variation’, loads on pitch variation and jitter. Factor 7, ‘MFCC 3’, loads on MFCC 3. In Supplementary Methods 3, we show the robustness of the factor solution across the largest countries and languages.

### Multilevel models

All multilevel models were fitted using the R package brms^72^, which is a high-level interface to Stan^73^. The models use the categorical response distribution and logit link function. Where possible, standard normal priors are used (that is, a normal distribution with a mean of 0 and a standard deviation of 1). The target distribution is explored using Hamiltonian Monte Carlo. The target acceptance rate is set to 99% to avoid divergent transitions after warmup. To avoid exceeding the maximum tree depth, we set the hyperparameter to 12. For reproducibility, all models use the same seed. To speed up sampling, we used cmdstan as a backend. All models use eight chains, and we collected 4,000 posterior samples. The models reported in the paper were defined as follows:

- Corpus model: emotion ~ 1 + RC1 + RC2 + RC3 + RC4 + RC5 + RC6 + RC7 + (1 + RC1 + RC2 + RC3 + RC4 + RC5 + RC6 + RC7 ∣ corpus)
- Null model: emotion ~ 1 + (1 ∣ corpus)
- Base model: emotion ~ 1 + RC1 + RC2 + RC3 + RC4 + RC5 + RC6 + RC7 + (1 ∣ corpus)
- Country model: emotion ~ 1 + RC1 + RC2 + RC3 + RC4 + RC5 + RC6 + RC7 + (1 + RC1 + RC2 + RC3 + RC4 + RC5 + RC6 + RC7 ∣ country) + (1 ∣ corpus)
- Language model: emotion ~ 1 + RC1 + RC2 + RC3 + RC4 + RC5 + RC6 + RC7 + (1 + RC1 + RC2 + RC3 + RC4 + RC5 + RC6 + RC7 ∣ language) + (1 ∣ corpus)
- Culture model: emotion ~ 1 + RC1 + RC2 + RC3 + RC4 + RC5 + RC6 + RC7 + (1 + RC1 + RC2 + RC3 + RC4 + RC5 + RC6 + RC7 ∣ country:language) + (1 ∣ corpus)
- Big model: emotion ~ 1 + RC1 + RC2 + RC3 + RC4 + RC5 + RC6 + RC7 + (1 + RC1 + RC2 + RC3 + RC4 + RC5 + RC6 + RC7 ∣ sex + country:language + speaker) + (1 ∣ corpus)

### SVMs

All SVM analyses reported in this paper were performed in Python and used the implementation from scikit-learn^74^. Following an INTERSPEECH challenge convention^75^, all SVMs use a linear kernel with the following complexities: 1 × 10^−5^, 1 × 10^−4^, 1 × 10^−3^, 1 × 10^−2^, 1 × 10^−1^ and 1.

### Heterogeneity index

To compute the *I*^2^ metric, we treated the model estimates for all corpora for the same emotion and acoustic factor as separate studies. First, we computed Conchran’s *Q* statistic, which is defined as:

$$Q=\sum {w}_{i}{({y}_{i}-\bar{\mu })}^{2}$$

where *i* is the index of the current corpus, *w*_*i*_ is the inverse variance of estimates of the current corpus, *y*_*i*_ is the mean estimate of the global mapping on top of the mapping of the current corpus and $\bar{\mu }$ is the weighted average over all corpora, defined as:

$$\frac{\sum {w}_{i}{\hat{y}}_{i}}{\sum {w}_{i}}$$

where ${\hat{y}}_{i}$ is the average estimate for the corpus alone.

Higgins and Thompson’s *I*^2^ is the percentage of variability in the effect sizes that is not caused by sampling error and is computed by:

$$\max \left(0,\frac{(Q/k-1)-1}{Q/k-1}\right)$$

where *k* is the number of corpora included in the analysis.

### Generalization analysis

To obtain the contributions of different levels of analysis to the prediction of the model, we first obtained the model prediction. For the predicted emotion, we summed all absolute values that go into the prediction for the emotion and divided each of the absolute values by this sum. This returns a contribution of single model parameters to the model prediction, which are each uniquely associated with one level of the analysis.

### Reporting summary

Further information on research design is available in the Nature Portfolio Reporting Summary linked to this article.

## Supplementary information

Supplementary Information Supplementary Figs. 1–7, Tables 1–4 and Discussion.

Reporting Summary

## Data Availability

The corpora used in this study are listed here: emotional.speechcorpora.com. The corpora of Adigwe et al.^66^, Burkhardt et al.^52^, Cao et al.^49^, Gournay et al.^48^, Haq and Jackson^62^, Livingstone and Russo^61^, Martin et al.^56^, and Pichora-Fuller and Dupuis^65^ can be downloaded directly. For the other corpora, we indicate how to contact the authors of the corpus on the website.

## Code availability

The code is stored on https://github.com/polvanrijn/mapping-nhb.
