# Supplementary material for: Modelling individual and cross-cultural variation in the mapping of emotions to speech prosody
Source: Nat Hum Behav. 2023 Jan 16;7(3):386–96. doi: 10.1038/s41562-022-01505-5 (PMC10038802; doi:10.1038/s41562-022-01505-5)
Supplement: Supplementary file 1 — Supplementary Figs. 1–7, Tables 1–4 and Discussion. [file 41562_2022_1505_MOESM1_ESM.pdf]

nature human behaviour

Article

https://doi.org/10.1038/s41562-022-01505-5

Modelling individual and cross-cultural variation in the mapping of emotions to speech prosody

In the format provided by the authors and unedited

Supplementary Methods

Corpus selection procedure

To query all available corpora capturing emotional prosody, we combined three search strategies with a final manual selection procedure (see Fig. 1 for an illustration). Each corpus included in the list needs to: (i) contain an annotation of the intended emotion per fragment, and (ii) consist of recordings of sentences (i.e., not a syllable, nonverbal vocalization, a phoneme, or a word).

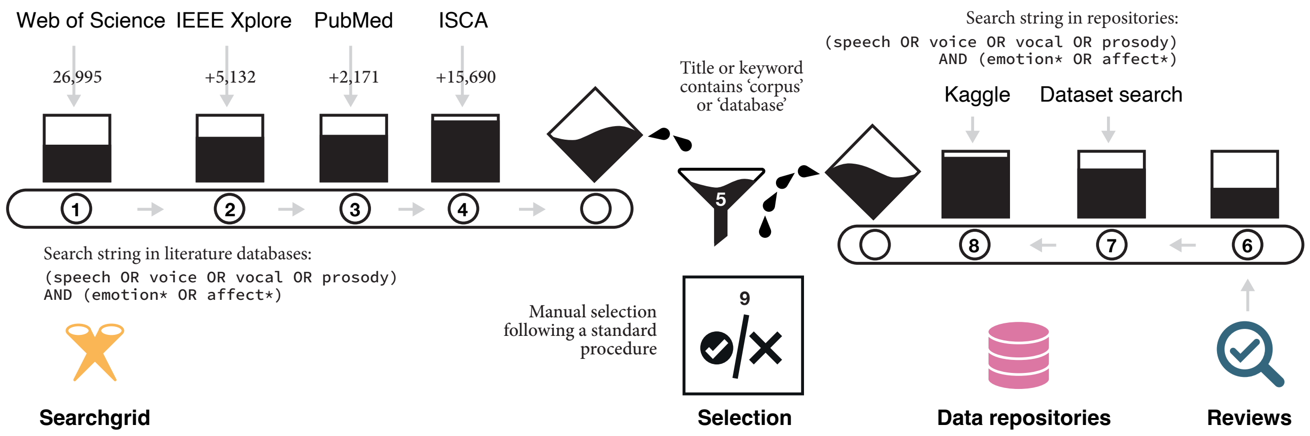

Supplementary Figure 1 | Procedure of collecting speech prosody corpora. Steps 1–4: compile a list of potential corpora by querying large literature search engines. Step 5: reduce the total number of potential corpora by filtering them either by title or keyword. Step 6: Extract potential corpora from existing literature reviews. Steps 7–8: Query data repositories. Step 9: Manual selection of corpora.

In the first approach (steps 1–4 in Fig. 1), we queried existing databases with a fixed set of keywords. We adapted an existing search query[1] — `(speech OR voice OR vocal OR prosody) AND (emotion* OR affect*)` — to query four databases: *PubMed*, *Web of Science* and *IEEE explore*. Since *PubMed* did not support wildcards, we omitted the asterisk (*) for this database. We did not include *PsycArticles*, as it does not allow us to automatically query the database, and *Google Scholar*, because search results are individualized to location and user. Since some corpora might only be announced at conferences, and conference proceedings are not always listed in databases, we also scanned all conferences organized by the ISCA, which is responsible for putting on the largest speech conference (INTERSPEECH). The results were not filtered by publication date. All database queries were performed on April 1st, 2020. This yields a large list of potential corpora that was further reduced. To avoid duplicate entries, we only included papers using a valid identifier (e.g., ISBN, DOI, ISCA-URL, or an identifier in the database). The long list ($\sim$ 50,000) was then filtered with the following criteria (step 5 in Fig. 1): either the title or one of the keywords contained the words "database" or "corpus" (case insensitive). Keywords were provided one of the following: by authors, the journal, or by the database. This allowed us to reduce the number of publications to 969, which were all checked by hand. In a second step, we scanned existing reviews of corpora containing emotional prosody[2–8] (step 6 in Fig. 1). As we might have missed some corpora in literature search engines, we also queried the data repositories *Kaggle* and *Google Dataset* (step 7–8 in Fig. 1). All potential corpora were manually checked using the predefined criteria described in the first paragraph (step 9 in Fig. 1). The remaining 24 corpora we obtained access to are listed in Tab. 2.

Corpus annotation

As depicted in Fig. 2, all annotations are centered around a single fragment. A fragment is spoken by a single speaker ( ), which has a biological sex ( ). In a given fragment, the speaker speaks a sentence ( ) in a specific language ( ) in a specific country ( ). For each fragment, it is clear which emotion was intended. If the same sentence is repeated by the same speaker for the same intended emotion, we call this a repetition ( ). Each fragment is part of a speech corpus ( ). For each corpus, we annotate if the speaker was also recorded on video ( ) and if the corpus is fully-crossed or not ( ). This means that the same sentence is produced in all emotions and thus implies that sentences in fully-crossed corpora are either semantically neutral or pseudo-speech. Pseudo-speech ( ) means that the fragment contains a Jabberwocky sentence, created with the phonological and syntactic characteristics of the reference language.

Then there are a series of annotations that are likely to be imperfect (marked by the warning sign in Fig. 2). The emotion induction procedure ( ) indicates how the emotion was elicited, which is either done by the meaning of the sentence, the emotion label (with description), a scenario, or a dyadic interaction. The exact procedure was not always described in the respective papers. Furthermore, emotion intensity ( ) indicates if the emotion intensity was experimentally manipulated in the fragment (yes or no). This annotation is impaired, as in most corpora, the emotion intensity was not explicitly controlled and thus undefined. The moderator "speaker type" ( ) describes if the speaker is a professional actor, an actor, or a speaker. The issue here is that each professional actor is also just an actor, and each actor is also a speaker. This circular structure makes the moderator suboptimal. We also annotate the year of publication or the release year of the corpus ( ) as a proxy for the year the stimuli were recorded in. Obviously, the delay between recording and publication is not always equally long, which

makes it a poor quality moderator. Finally, we also annotated if the corpus was 'validated' (✅), however details about the validation (e.g. number of raters per fragment, exact paradigm) are not always described in the papers, which makes the moderator not very informative.

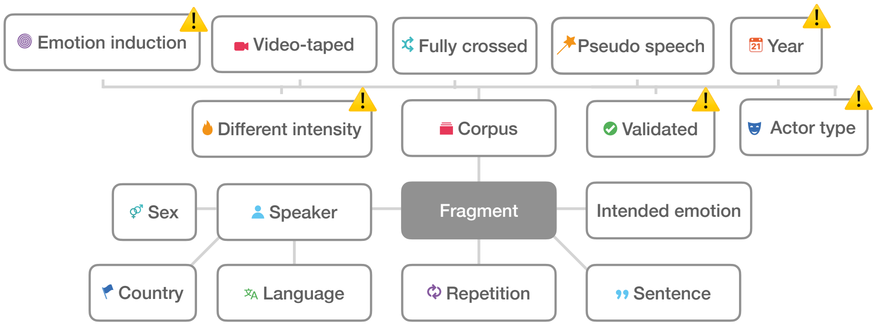

Supplementary Figure 2 | Relationship between the extracted annotations per corpus. All annotations are centered around a single recording — the "fragment". The warning sign indicates that the quality of the moderator is impaired, as described in the main text.

Factor analysis and robustness of the solution

To interpret coefficients in a regression, the number of predictors should be limited to a set of relevant features and the correlations among predictor variables should be low. Strong correlations among predictors — also called multicollinearity — inflate the coefficients of the predictors.

One approach to reduce the amount of features is to select relevant predictors based on previous findings. However, acoustic features are correlated with each other (e.g., see Fig. 3a). Using bootstrapping, we randomly select seven features from the eGeMAPS feature set (n = 10,000). As depicted in Fig. 3b, there is a large probability that at least one pair of predictors is strongly correlated (notice the peaks in the distribution of the correlations at .75, .45, or .95). Moreover, only a small proportion of seven features picked at random have a maximum correlation that is low ($r < .30$).

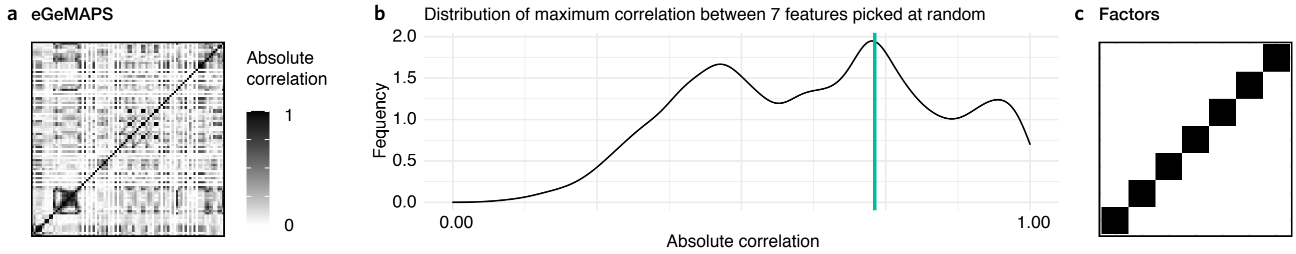

Supplementary Figure 3 | Correlation among acoustic features. a Correlations between the 88 features in the eGeMAPS set. b Distribution of maximum absolute correlation between one pair of variables when randomly selecting 7 eGeMAPS features (n = 10,000). The vertical green line indicates the largest peak in the distribution. **c** Correlations between seven factors with Varimax rotation. Same legend as in **a**.

Feature selection is not a solution, as the correlation among at least one pair of the features would be too high and thus impair the interpretation of the model. An alternative to feature selection is dimension reduction, which yields orthogonal (i.e., uncorrelated) dimensions. However, dimension reduction can come at the price of interpretability: resulting dimensions often contain blends of variables, and selected features usually capture one aspect of the signal (e.g., loudness). An intermediate position is taken by factor analysis with varimax rotation. Factor analysis enables a reduction in a set of observed, correlated variables to a potentially lower number of latent variables called "factors". The varimax rotation allows the factors to have a very low/no correlation among each other (see Fig. 3c).

We initially computed the factor analysis on a balanced subset of the corpora, indicating each emotion occurred equally often and thus had an equal contribution to the factor solution. Here, the objective was to identify a minimal amount of factors and to keep the model as simple as possible, while still having decent predictive accuracy. To address this issue, we ran a series of SVMs that predict the emotions in each corpus separately for an increasing number of factor solutions (four-fold leave-speaker-out cross-validation if possible, identical hyperparameters as in the main paper). Since the corpora included in the analysis consist of a different number of stimuli, we worried that large corpora might dominate the factor analysis. We, therefore, did not only compute the factor solutions on all corpora at once but also on each corpus separately. We obtained the mean unweighted average recall (UAR) across corpora for an increasing number of factors. As depicted in Fig. 4a, both the UAR for the dimension reduction across all corpora or within a corpus increase as more factors are added. For factor solutions 3–6, we can see the UAR is larger (note the non-overlapping CIs) for the dimension reduction per corpus compared to the dimension reduction on all corpora at once. However, for factor solutions with seven or more factors, there is no structural advantage to applying the dimension reduction on a corpus level. In a second analysis, we fitted a series of Bayesian multinomial logistic regressions using 1 up to 10 factors. As depicted in Fig. 4b, the WAIC improvement stagnates for factor solutions with more than seven features.

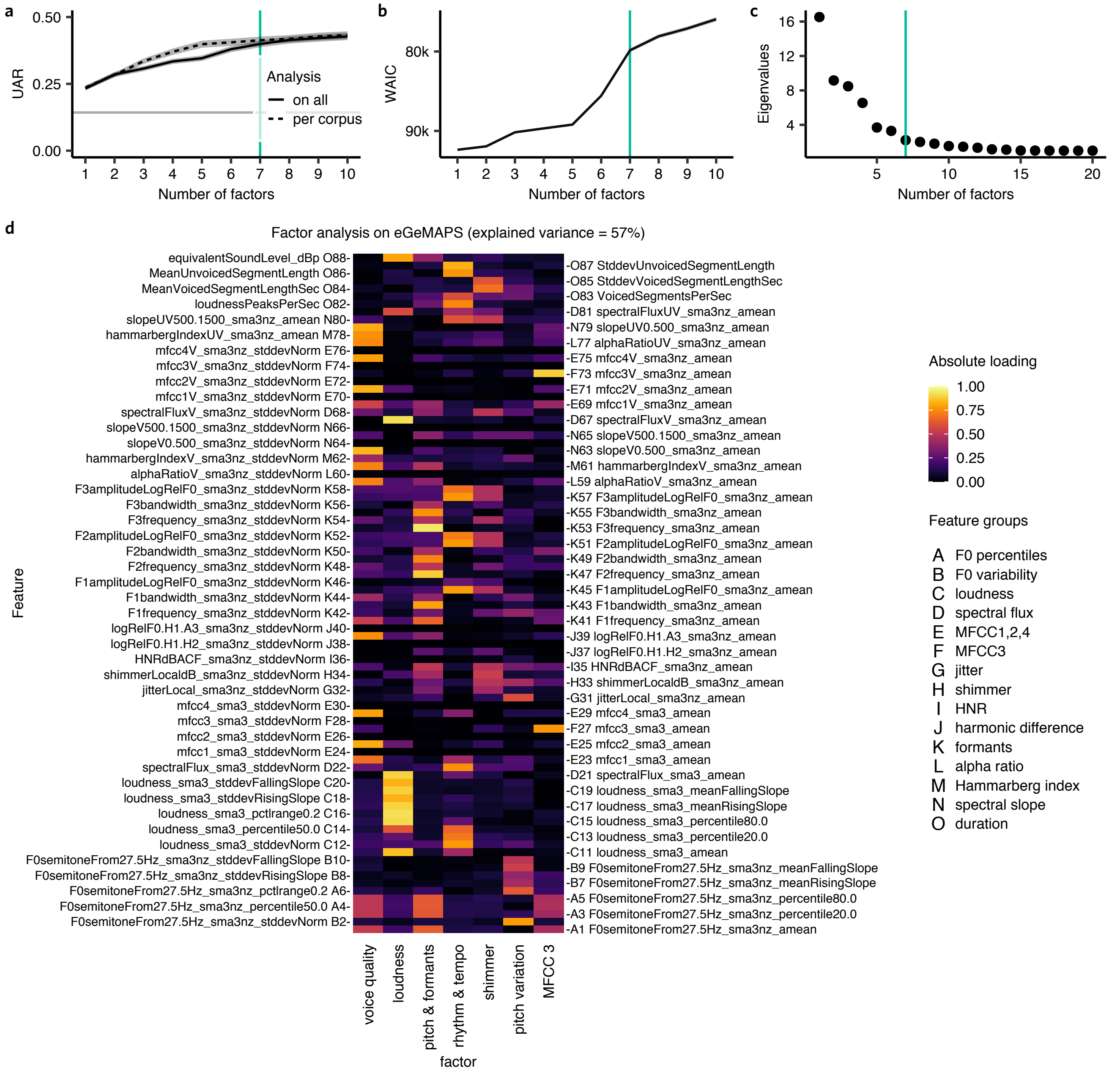

Supplementary Figure 4 | Factor analysis. Justification of the number of factors and loading plot. **a** Unweighted average recall (UAR) for an increasing number of factor solutions. The dashed line indicates the mean UAR for factor solutions computed on each corpus separately and the straight line the factor solutions on all corpora at once. The area around the lines is a 95% confidence interval. The horizontal line indicates chance level (i.e. 14.3%). **b** WAIC for models with an increasing number of factors. The dark area around the line is an 89% credible interval. **c** Screeplot for factor solution on all 24 corpora. **d** Loading plot for the solution on all 24 corpora. The eGeMAPS features are grouped into feature groups. The fill color is the absolute loading on the factor.

Type	Acoustic cue	Definition and measurement	Perceived correlate
Frequency	Fundamental frequency (F_0)	F_0 describes the rate of vibration of the vocal folds. It is described with summary statistics (e.g., arithmetic mean). The change of F_0 over time (referred to as pitch contour) is solely described with a slope. F_0 tends to be higher in aroused states[33].	Pitch and intonation contour
	Jitter	Jitter refers to small perturbations in F_0 in one cycle to another. It is caused by irregular fluctuations in the time it takes to open and close the vocal folds.	Pitch perturbations; “roughness” in the voice[34]
	First three formants (F_1 to F_3)	Caused by resonance in and speaker modulations of the vocal tract.	Voice quality[35]
Amplitude	Intensity	Sum of amplitudes across all frequency bands. It reflects the effort of the speaker to produce the utterance. Another amplitude measure used is equivalent sound level, which expresses the amplitude in decibels.	Loudness of speech
	Shimmer	Variations in amplitude from cycle to cycle, caused by irregular fluctuations in amplitude.	”Roughness” in the voice[36]
	Harmonics-to-noise ratio (HNR)	Proportion between harmonic (e.g., in vowels) and noise components (e.g., in unvoiced speech) in the voice.	”Breathy voice”[37]
Spectral balance	Alpha ratio	Ratio between the summed amplitude in the 50-1000 Hz and 1-5 kHz frequency bands.	Voice quality[38]
	Hammarberg index	Ratio of the strongest peak amplitude in the 0-2 kHz and the 2–5 kHz frequency bands.	Vocal effort[39]
	Spectral slope	Linear regression slope of the amplitudes of two frequency bands 0-500Hz, 500-1500Hz.	Voice quality[40]
	Energy proportion	Energy below and above 500 Hz, and 1000 Hz respectively.	Voice quality (related to spectral slope)[40]
	Harmonic difference	Difference H1 and H2 and H1 and A3, where the first F_0 harmonic is H1, and the second harmonic is H2; A3 is the highest harmonic in the third formant range.	Voice quality (also related to spectral slope)[40]
	Relative energy in F_{1-3}	Amplitude of the formants relative to F_0	Voice quality[35]
	Spectral flux	Speed at which energy distribution in different frequencies changes over time.	Rhythm and timbre[41]
	MFCCs (1–4)	Mel Frequency Cepstral Coefficients using the Mel frequency scale which mimics human hearing.	Timbre[42]
Duration	Rate of loudness peaks	Number of loudness peaks per second.	Velocity of speech
	Number voiced regions per second	Number of continuous voiced regions per second; similar to syllable rate.	Velocity of speech
	Duration of (un-) voiced regions	Duration of consecutive voiced or unvoiced regions; unvoiced regions approximate pauses.	Speech rhythm and fluency

Supplementary Table 1 | Description of the features included in the eGeMAPS feature set. Also, see the summary in Nordström *et al.* [43].

.

Name	Citation	N	Duration	Emotion	Language	Country	Speaker (fem.)	Pseudo-speech	Fully crossed	Different intensity	Allow rep.	Video recorded	Ind. proc.	Speaker type	Validated
AHOEMO1	Navas *et al.* [9]	1133	84	ANG, DIS, FER, HAP, NEU, SAD, SUR	EU	ES	1(1)	0	0	0	0	0	-	1	0
AHOEMO2	Saratxaga *et al.* [10]	9828	1040	ANG, DIS, FER, HAP, NEU, SAD, SUR	EU	ES	2(1)	0	0	0	0	0	1	1	0
CaFE	Gournay *et al.* [11]	936	69	ANG, DIS, FER, HAP, NEU, SAD, SUR	FR	CA	12(6)	0	1	1	0	0	2	2	1
CREMA-D	Cao *et al.* [12]	7442	315	ANG, DIS, FER, HAP, NEU, SAD	EN	US	91(43)	0	1	0	0	1	3	1	1
DaFEX	Battocchi *et al.* [13]	285	44	ANG, DIS, FER, HAP, NEU, SAD, SUR	IT	IT	8(4)	0	1	1	1	1	3	1	0
DB Arabic	Hadjadji *et al.* [14]	5344	134	ANG, HAP, NEU, SAD	AR	DZ	13(7)	0	1	0	1	0	-	3	0
EMO-DB	Burkhardt *et al.* [15]	535	24	ANG, DIS, FER, HAP, NEU, SAD, BOR	DE	DE	10(4)	0	1	0	1	0	3	2	1
EEKK	Altrov & Pajupuu [16]	1106	61	ANG, HAP, NEU, SAD	ET	EE	2(2)	0	0	0	0	0	1	3	1
EmoHI	Nagels *et al.* [17]	94	3	ANG, HAP, NEU, SAD	NL	NL	6(3)	1	1	0	1	0	-	3	1
EMA	Lee *et al.* [18]	680	51	ANG, HAP, NEU, SAD	EN	US	3(2)	0	1	0	0	0	-	3	0
eNTERFACE	Martin *et al.* [19]	1287	60	ANG, DIS, FER, HAP, SAD, SUR	EN	BE*	43(9)	0	0	0	0	1	3	3	0
ESCAD	Ykhlef *et al.* [20]	2527	64	ANG, NEU	AR	DZ	53(28)	0	0	0	1	0	2	3	1
GEMEP	Bänziger *et al.* [21]	931	39	DIS, SAD, AMU, ADM, ANX, CAN, CON, DES, ELA, HAN, INT, PAN, PLE, PRI, REL, SHA, TEN	FR	CH	10(5)	1	1	0	1	0	3	1	1
MAP HAWK	Hawk *et al.* [22]	80	4	ANG, DIS, FER, HAP, NEU, SAD, SUR, CON, PRI, EMB	NL	NL	8(4)	0	1	0	0	1	2	1	1
MSP- improv	Busso *et al.* [23]	1272	90	ANG, HAP, NEU, SAD	EN	US	12(6)	0	0	0	1	1	4	2	1
-	Juslin & Laukka [24]	176	5	ANG, DIS, FER, HAP, NEU, SAD	SV, EN	SE	8(4)	0	1	1	0	0	2	1	1
-	Pell *et al.* [25]	1602	46	ANG, DIS, FER, HAP, NEU, SAD, SUR	EN	CA	4(2)	1**	1	0	1	0	2	2	1
RAVDESS	Livingstone & Russo [26]	1440	88	ANG, DIS, FER, HAP, NEU, SAD, SUR, CAL	EN	CA	24(12)	0	1	1	1	1	2	1	1
SAVEE	Haq & Jackson [27]	480	30	ANG, DIS, FER, HAP, NEU, SAD, SUR	EN	GB	4(0)	0	0	0	0	1	2	2	1
IITKGP-SESC	Koolagudi *et al.* [28]	12000	406	ANG, DIS, FER, HAP, NEU, SAD, SUR, SAR	TE	IN	7(4)†	0	1	0	1	0	2	1	1
IITKGP-SEHSC	Koolagudi *et al.* [29]	7133	578	ANG, DIS, FER, HAP, NEU, SUR, SAR, COM	HI	IN	10(5)	0	1	0	1	0	2	1	1
TESS	Pichora-Fuller & Dupuis [30]	2800	95	ANG, DIS, FER, HAP, NEU, SAD, SUR	EN	CA	2(2)	0	1	0	0	0	-	2	0
EmoV-DB	Adigwe *et al.* [31]	6580	541	ANG, DIS, NEU, AMU, SLE	EN	US	4(2)	0	1	0	0	0	2	2	0
VENEC	Laukka *et al.* [32]	550	16	ANG, FER, HAP, NEU, SAD, CON, PRI, REL, SHA, INT, LUS	EN	AU, IN, KE, SG, US	95(48)	0	1	0	0	0	3	1	1

Supplementary Table 2 | List of all corpora included in the analysis. *Name* = short name of the corpus given by the authors, *Citation* = the publication of the corpus, *N* = number of fragments per corpus, *Duration* = total duration in minutes, *Language* = ISO 639-1 language code, *Country* = ISO 3166 country code, *Speaker* = number of speakers (number of females in brackets), *Pseudo-speech* = corpus consists of nonsense sentences, *Fully crossed* = all sentences are recorded for all emotions, *Different intensity* = emotional intensity was experimentally manipulated, *Allow rep.* = same sentence emotion pair may be recorded more than once, *Video recorded* = participants were videotaped while producing the sentences, *Ind. proc.* (Induction procedure) = the type of emotion induction approach (1 = by the meaning of the sentence, 2 = emotion label (with description), 3 = scenario, 4 = dyadic interaction), *Speaker type* = either professional actor if explicitly stated in the manuscript (= 1), actor if it was unclear if professional or amateur (=2) and non-actor (= 3). *Validated* = yes (1) or no (0). * eNTERFACE was recorded during a conference, the country of participants could not be obtained. ** 50% of the corpus is pseudo-speech the other 50% consist of regular sentences. † The authors report 10 speakers, but in the data received from the authors there are only 7 speakers.

A factor analysis is now computed on all 24 corpora. Prior to the analysis, all data was standardized to have zero mean and unit variance. The means are reported in Tables 3 and 4. There is a strong average correlation (.86) between the factor solutions on the balanced and all data. We, therefore, use factor analysis on all data. While it remains debatable, why exactly seven factors and not some more or less were used (compare scree plot in Fig. 4c), the loading plot (Fig. 4d) indicates that the seven factors load on different prosodic dimensions that are perceptually relevant for the communication of emotion (see Tab. 1 for the perceived correlates of the eGeMAPS features).

To further assess the robustness of the factor analysis, we compute a seven solution factor analysis for each of the four largest countries and largest languages, covering 87% and 89% of the data, respectively. We predict all data into the factor analysis of the respective country or language. For each country and each language pair, we compute the optimal alignment, by maximizing the correlation between the dimensions. For each country and language pair, we compute an average correlation of each of the seven aligned factors. Some country and language pairs align better with each other, but on average we find a correlation of $r = .67$ and $r = .65$ indicating a fair overlap in factor solutions across languages and countries (see Fig. 5).

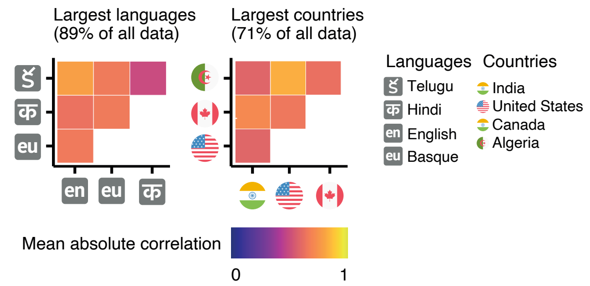

Supplementary Figure 5 | Correlation among factor solutions for the four largest languages and countries. The factor solutions are relatively robust across the most common countries (on average $r = .67$) and languages ($r = .65$) in the datasets.

Feature	Mean
F0semitoneFrom27.5Hz_sma3nz_amean	33.5810
F0semitoneFrom27.5Hz_sma3nz_stddevNorm	0.103000
F0semitoneFrom27.5Hz_sma3nz_percentile20.0	31.0130
F0semitoneFrom27.5Hz_sma3nz_percentile50.0	33.6190
F0semitoneFrom27.5Hz_sma3nz_percentile80.0	36.2440
F0semitoneFrom27.5Hz_sma3nz_pctlrange0.2	5.23100
F0semitoneFrom27.5Hz_sma3nz_meanRisingSlope	103.870
F0semitoneFrom27.5Hz_sma3nz_stddevRisingSlope	100.385
F0semitoneFrom27.5Hz_sma3nz_meanFallingSlope	48.0320
F0semitoneFrom27.5Hz_sma3nz_stddevFallingSlope	46.3680
loudness_sma3_amean	0.594000
loudness_sma3_stddevNorm	0.880000
loudness_sma3_percentile20.0	0.148000
loudness_sma3_percentile50.0	0.443000
loudness_sma3_percentile80.0	1.01900
loudness_sma3_pctlrange0.2	0.871000
loudness_sma3_meanRisingSlope	10.7260
loudness_sma3_stddevRisingSlope	5.91200
loudness_sma3_meanFallingSlope	9.73200
loudness_sma3_stddevFallingSlope	5.23700
spectralFlux_sma3_amean	0.319000
spectralFlux_sma3_stddevNorm	1.06100
mfcc1_sma3_amean	18.0420
mfcc1_sma3_stddevNorm	1.00900
mfcc2_sma3_amean	7.04100
mfcc2_sma3_stddevNorm	0.717000
mfcc3_sma3_amean	8.52800
mfcc3_sma3_stddevNorm	-0.461000
mfcc4_sma3_amean	-3.10900
mfcc4_sma3_stddevNorm	0.926000
jitterLocal_sma3nz_amean	0.0330000
jitterLocal_sma3nz_stddevNorm	1.12000
shimmerLocaldB_sma3nz_amean	1.23600
shimmerLocaldB_sma3nz_stddevNorm	0.815000

HNRdBACF_sma3nz_amean	7.25000
HNRdBACF_sma3nz_stddevNorm	0.446000
logRelF0.H1.H2_sma3nz_amean	7.18500
logRelF0.H1.H2_sma3nz_stddevNorm	1.85200
logRelF0.H1.A3_sma3nz_amean	20.7250
logRelF0.H1.A3_sma3nz_stddevNorm	0.930000
F1frequency_sma3nz_amean	634.089
F1frequency_sma3nz_stddevNorm	0.262000
F1bandwidth_sma3nz_amean	1384.57
F1bandwidth_sma3nz_stddevNorm	0.152000
F1amplitudeLogRelF0_sma3nz_amean	-111.301
F1amplitudeLogRelF0_sma3nz_stddevNorm	-0.873000
F2frequency_sma3nz_amean	1576.42
F2frequency_sma3nz_stddevNorm	0.124000
F2bandwidth_sma3nz_amean	1067.35
F2bandwidth_sma3nz_stddevNorm	0.257000
F2amplitudeLogRelF0_sma3nz_amean	-111.420
F2amplitudeLogRelF0_sma3nz_stddevNorm	-0.798000
F3frequency_sma3nz_amean	2528.97
F3frequency_sma3nz_stddevNorm	0.0710000
F3bandwidth_sma3nz_amean	932.149
F3bandwidth_sma3nz_stddevNorm	0.305000
F3amplitudeLogRelF0_sma3nz_amean	-113.575
F3amplitudeLogRelF0_sma3nz_stddevNorm	-0.751000
alphaRatioV_sma3nz_amean	-13.6010
alphaRatioV_sma3nz_stddevNorm	-0.662000
hammarbergIndexV_sma3nz_amean	23.0040
hammarbergIndexV_sma3nz_stddevNorm	0.393000
slopeV0.500_sma3nz_amean	0.0380000
slopeV0.500_sma3nz_stddevNorm	0.680000
slopeV500.1500_sma3nz_amean	-0.0140000
slopeV500.1500_sma3nz_stddevNorm	-1.22800
spectralFluxV_sma3nz_amean	0.501000
spectralFluxV_sma3nz_stddevNorm	0.640000
mfcc1V_sma3nz_amean	25.2950
mfcc1V_sma3nz_stddevNorm	0.444000
mfcc2V_sma3nz_amean	3.01100
mfcc2V_sma3nz_stddevNorm	54.3800
mfcc3V_sma3nz_amean	9.24700
mfcc3V_sma3nz_stddevNorm	0.545000
mfcc4V_sma3nz_amean	-8.97200
mfcc4V_sma3nz_stddevNorm	-0.918000
alphaRatioUV_sma3nz_amean	-11.4120
hammarbergIndexUV_sma3nz_amean	20.3860
slopeUV0.500_sma3nz_amean	0.00900000
slopeUV500.1500_sma3nz_amean	0.00500000
spectralFluxUV_sma3nz_amean	0.164000
loudnessPeaksPerSec	3.72600
VoicedSegmentsPerSec	2.28500
MeanVoicedSegmentLengthSec	0.239000
StddevVoicedSegmentLengthSec	0.148000
MeanUnvoicedSegmentLength	0.245000
StddevUnvoicedSegmentLength	0.245000
equivalentSoundLevel_dBp	-24.6150

Supplementary Table 3 | Average feature values for eGeMAPS.

Feature	Anger	Disgust	Fear	Happiness	Sadness	Surprise	Neutral
alphaRatioUV_sma3nz_amean	-10.3464	-11.4382	-11.8240	-11.6069	-12.5854	-9.92991	-12.9356
alphaRatioV_sma3nz_amean	-10.0484	-12.7334	-14.9715	-13.1112	-18.0043	-13.3013	-15.3181

Feature	Anger	Disgust	Fear	Happiness	Sadness	Surprise	Neutral
alphaRatioV_sma3nz_stddevNorm	-1.02659	-0.618631	-0.513823	-0.949250	-0.446089	-0.665625	-0.530521
equivalentSoundLevel_dBp	-21.6667	-25.9564	-25.1559	-23.7905	-28.2641	-23.4758	-26.3597
F0semitoneFrom27.5Hz_sma3nz _amean	36.2483	31.5199	34.0638	34.4284	30.8265	36.4274	31.0419
F0semitoneFrom27.5Hz_sma3nz _meanFallingSlope	48.0418	49.5392	42.4309	50.7773	42.4249	67.7286	37.1470
F0semitoneFrom27.5Hz_sma3nz _meanRisingSlope	101.988	106.303	92.9059	94.4739	118.574	98.2575	94.6538
F0semitoneFrom27.5Hz_sma3nz _pctlrange0.2	5.30119	4.92544	3.94585	6.55041	4.13090	7.99285	4.51986
F0semitoneFrom27.5Hz_sma3nz _percentile20.0	33.6845	29.0676	32.1392	31.2246	28.8781	32.4236	28.8162
F0semitoneFrom27.5Hz_sma3nz _percentile50.0	36.4155	31.4586	34.1855	34.2957	30.9349	36.5484	30.9813
F0semitoneFrom27.5Hz_sma3nz _percentile80.0	38.9856	33.9931	36.0850	37.7750	33.0090	40.4164	33.3360
F0semitoneFrom27.5Hz_sma3nz _stddevFallingSlope	47.9128	48.2392	39.7114	46.2204	40.0351	69.0252	33.2303
F0semitoneFrom27.5Hz_sma3nz _stddevNorm	0.0991567	0.106707	0.0832259	0.114883	0.0998071	0.127049	0.0993584
F0semitoneFrom27.5Hz_sma3nz _stddevRisingSlope	101.856	106.637	97.3278	87.9271	108.610	84.5611	90.0090
F1amplitudeLogRelF0_sma3nz _amean	-104.677	-116.268	-120.128	-109.633	-133.405	-106.336	-107.369
F1amplitudeLogRelF0_sma3nz _stddevNorm	-0.936232	-0.829330	-0.771866	-0.879072	-0.665881	-0.897900	-0.904798
F1bandwidth_sma3nz_amean	1388.16	1399.81	1365.14	1380.33	1394.49	1365.93	1398.26
F1bandwidth_sma3nz_stddevNorm	0.164578	0.144430	0.152688	0.149734	0.142819	0.162058	0.141132
F1frequency_sma3nz_amean	682.352	608.437	637.997	641.305	608.878	669.045	598.886
F1frequency_sma3nz_stddevNorm	0.263955	0.265451	0.260778	0.256087	0.258789	0.263992	0.254261
F2amplitudeLogRelF0_sma3nz _amean	-101.651	-117.521	-118.800	-109.665	-135.439	-104.902	-110.739
F2amplitudeLogRelF0_sma3nz _stddevNorm	-0.901206	-0.775081	-0.717812	-0.800468	-0.602962	-0.824833	-0.789497
F2bandwidth_sma3nz_amean	1089.59	1077.70	1054.51	1055.51	1074.54	1075.51	1059.25
F2bandwidth_sma3nz_stddevNorm	0.257930	0.253124	0.251624	0.256451	0.256276	0.261747	0.256662
F2frequency_sma3nz_amean	1601.88	1547.79	1568.44	1585.97	1583.61	1604.09	1562.90
F2frequency_sma3nz_stddevNorm	0.122462	0.124072	0.120978	0.119220	0.128632	0.123800	0.127982
F3amplitudeLogRelF0_sma3nz _amean	-103.719	-119.445	-120.806	-111.653	-137.558	-107.085	-113.323
F3amplitudeLogRelF0_sma3nz _stddevNorm	-0.849115	-0.729986	-0.677089	-0.759944	-0.568556	-0.774840	-0.739383
F3bandwidth_sma3nz_amean	929.453	939.697	913.122	938.192	949.097	950.850	930.616
F3bandwidth_sma3nz_stddevNorm	0.298182	0.296962	0.296610	0.300685	0.307733	0.314277	0.314756
F3frequency_sma3nz_amean	2544.82	2489.42	2517.11	2533.50	2556.69	2539.14	2539.77
F3frequency_sma3nz_stddevNorm	0.0692871	0.0726109	0.0673270	0.0683218	0.0755249	0.0720046	0.0733199
hammarbergIndexUV_sma3nz _amean	19.3980	20.3887	20.6538	20.3991	21.6831	18.7761	21.9827
hammarbergIndexV_sma3nz _amean	19.3758	21.9943	24.3559	22.1952	28.0205	22.9798	24.7953
hammarbergIndexV_sma3nz _std-devNorm	0.480932	0.415456	0.351908	0.396819	0.307823	0.405269	0.352421
HNRdBACF_sma3nz_amean	6.97629	6.29637	7.84323	7.23992	7.09577	7.81480	6.94745
HNRdBACF_sma3nz_stddevNorm	0.487651	0.522630	0.388423	0.445683	0.440203	0.450833	0.413988
jitterLocal_sma3nz_amean	0.0331365	0.0320741	0.0299928	0.0345058	0.0314539	0.0411424	0.0305484
jitterLocal_sma3nz_stddevNorm	1.11454	1.06614	1.02594	1.12799	1.12890	1.18890	1.08586
logRelF0.H1.A3_sma3nz_amean	17.4984	20.5780	21.3323	19.7186	25.7483	19.4195	23.4639
logRelF0.H1.A3_sma3nz_stddevNorm	0.733723	3.67242	0.497654	0.552177	0.459077	0.611981	0.417001
logRelF0.H1.H2_sma3nz_amean	6.85077	6.13753	8.39757	7.22909	7.23188	8.71647	6.07511
logRelF0.H1.H2_sma3nz_stddevNorm	2.63497	1.06725	0.775580	2.84387	1.54565	1.18482	2.62348

Feature	Anger	Disgust	Fear	Happiness	Sadness	Surprise	Neutral
loudness_sma3_amean	0.772661	0.569084	0.536269	0.641372	0.373815	0.625319	0.503581
loudness_sma3_meanFallingSlope	12.9356	9.11727	8.90069	10.7794	6.16282	10.8806	8.53949
loudness_sma3_meanRisingSlope	14.1517	9.98163	9.64083	11.9100	6.98431	11.6834	9.16166
loudness_sma3_pctlrange0.2	1.17293	0.832404	0.772812	0.992981	0.549301	0.919844	0.728085
loudness_sma3_percentile20.0	0.172829	0.138223	0.144499	0.143426	0.0951353	0.155704	0.126472
loudness_sma3_percentile50.0	0.564938	0.429408	0.408935	0.454859	0.260306	0.489942	0.373033
loudness_sma3_percentile80.0	1.34576	0.970627	0.917311	1.13641	0.644437	1.07555	0.854556
loudness_sma3_stddevFallingSlope	6.82918	4.94879	4.67585	5.57438	3.51277	5.51948	4.65368
loudness_sma3_stddevNorm	0.935428	0.889578	0.845422	0.903863	0.873846	0.894878	0.855947
loudness_sma3_stddevRisingSlope	7.41441	5.54338	5.38341	6.52694	4.23618	6.18944	4.95063
loudnessPeaksPerSec	3.81872	3.53046	3.80308	3.65006	2.99029	4.00075	3.95426
MeanUnvoicedSegmentLength	0.216175	0.280571	0.249790	0.234911	0.346942	0.207380	0.224872
MeanVoicedSegmentLengthSec	0.248384	0.233847	0.220649	0.244489	0.217274	0.228202	0.232285
mfcc1_sma3_amean	15.3076	17.8467	17.8300	18.7709	19.3394	16.1729	20.7996
mfcc1_sma3_stddevNorm	0.746431	0.590297	0.923265	0.977185	1.10093	0.743002	0.888580
mfcc1V_sma3nz_amean	19.9123	25.6000	24.2277	24.9174	30.0705	22.7734	29.8262
mfcc1V_sma3nz_stddevNorm	0.752599	0.425349	0.252574	0.419147	0.312216	0.526636	0.345671
mfcc2_sma3_amean	3.15504	7.71567	9.57936	6.35476	12.9232	5.37554	10.3314
mfcc2_sma3_stddevNorm	4.04233	1.36468	-0.109990	-0.736472	0.469147	-1.44383	-0.101269
mfcc2V_sma3nz_amean	-3.00208	3.65371	6.25717	1.14076	10.7918	1.76132	6.40704
mfcc2V_sma3nz_stddevNorm	-0.0414873	0.139127	1.62341	438.295	0.756565	-0.511039	0.873659
mfcc3_sma3_amean	5.00146	9.02361	9.12553	8.84246	10.8221	5.58173	10.5414
mfcc3_sma3_stddevNorm	1.66592	4.09892	0.310829	-1.22171	-15.5816	-4.95262	3.83739
mfcc3V_sma3nz_amean	4.36775	10.1374	9.42230	8.65490	12.3980	5.87783	11.8332
mfcc3V_sma3nz_stddevNorm	3.91104	0.387811	-1.71823	0.767225	5.35971	-3.27975	0.811149
mfcc4_sma3_amean	-7.86172	-1.39988	-1.36180	-3.65310	3.01221	-4.96738	-1.50533
mfcc4_sma3_stddevNorm	-2.14710	-0.0595681	4.88684	3.61135	2.40472	0.220496	0.136054
mfcc4V_sma3nz_amean	-15.8446	-7.09542	-6.57913	-10.2498	-2.29066	-10.3992	-6.95668
mfcc4V_sma3nz_stddevNorm	-1.30955	-1.22309	-0.869668	-2.09989	3.17359	-2.62762	-1.54347
shimmerLocaldB_sma3nz_amean	1.25382	1.25254	1.23824	1.23577	1.27729	1.26278	1.27737
shimmerLocaldB_sma3nz _stddevNorm	0.781846	0.772317	0.764285	0.805679	0.834223	0.800973	0.869127
slopeUV0.500_sma3nz_amean	0.0110221	0.0108024	0.0105526	0.0105148	-8.59171e-06	0.0224552	-0.00225137
slopeUV500.1500_sma3nz_amean	0.00462410	0.00497867	0.00537710	0.00453137	0.00755221	0.00721432	0.00467271
slopeV0.500_sma3nz_amean	0.0473562	0.0382995	0.0349388	0.0421471	0.0252936	0.0498223	0.0292241
slopeV0.500_sma3nz_stddevNorm	0.910005	-1.60569	3.62147	0.654498	0.826579	0.883494	0.436426
slopeV500.1500_sma3nz_amean	-0.0137875	-0.0140333	-0.0126989	-0.0155787	-0.0149801	-0.0120635	-0.0174340
slopeV500.1500_sma3nz_stddevNorm	-0.447016	-1.20596	-1.61011	-1.10170	-1.45695	-1.76130	-1.22107
spectralFlux_sma3_amean	0.436788	0.275499	0.300262	0.365209	0.201104	0.368570	0.252419
spectralFlux_sma3_stddevNorm	1.13333	1.05559	1.03126	1.08881	1.05783	1.09652	0.997574
spectralFluxUV_sma3nz_amean	0.208072	0.148032	0.165488	0.166065	0.117162	0.194745	0.126115
spectralFluxV_sma3nz_amean	0.684656	0.436995	0.476317	0.595390	0.342219	0.563045	0.404708
spectralFluxV_sma3nz_stddevNorm	0.692822	0.623660	0.586410	0.641138	0.595349	0.639608	0.611684
StddevUnvoicedSegmentLength	0.222391	0.265320	0.249843	0.245895	0.335361	0.236503	0.225671
StddevVoicedSegmentLengthSec	0.152958	0.148079	0.128627	0.149276	0.137485	0.146624	0.139471
VoicedSegmentsPerSec	2.41034	2.06338	2.29856	2.28933	1.96023	2.51165	2.46243

Supplementary Table 4 | Average feature values for eGeMAPS per emotion.

Model comparison

The Widely Applicable Information Criterion (WAIC)[44] is an information criterion that — in contrast to the Akaike Information Criterion (AIC) and Deviance Information Criterion — does not make an assumption about the shape of the posterior. As depicted in Equation 1, it consists of two parts: a log-pointwise-predictive-density estimate (lppd) and a penalty term. The difference between the two is multiplied by -2, which follows the same scaling convention as in AIC.

$$\mathrm{WAIC}(y, \Theta) = -2\big(\mathrm{lppd} - \mathrm{penalty}\big) \tag{1}$$

The lppd gives us the log probability score for each specific observation. Larger values indicate larger average accuracy. Following Equation 2, lppd takes the original data y and the posterior distribution Θ as an input. Here, i is the index of the current observation — in our case,

a single speech recording — and S is the total number of posterior samples (in our models always 4,000) with s as the index of the current sample. In other words: we compute the probability of each recording i for each posterior sample s, and then we take the average and the logarithm.

$$\text{lppd}(y, \Theta) = \sum_i \log \frac{1}{S} \sum_s p(y_i|\Theta_s) \tag{2}$$

We can observe that lppd keeps improving for increasingly complex models. This is unwanted behavior, since we want to identify which models are plausible given the data and not which models overfit most. To avoid this issue, a penalty term is introduced (see Equation 3).

$$\text{penalty}(y, \Theta) = \sum_i \text{var}_\theta \log p(y_i|\theta) \tag{3}$$

The penalty computes the variance in log-probabilities for each recording i and sums it up. The larger the variance in each recording, the more the model tends to overfit. Since we obtain a log-pointwise-predictive-density estimate for every recording, we can compute the standard error, which is defined in Equation 4, where N is the total amount of recordings.

$$\text{WAIC}_{\text{SE}} = \sqrt{N\text{var} - 2(\text{lppd}_i - \text{penalty}_i)} \tag{4}$$

Due to the pointwise nature of the WAIC, it is important to ascertain that the WAIC is not driven by a few extreme observations. The importance of single estimates can be estimated using Pareto-smoothed importance sampling cross-validation (short PSIS-loo). For each recording, a k value is estimated that provides information about the reliability of the approximation. Larger k values are more influential in the WAIC. Model comparisons using WAIC yielded identical results compared to PSIS-loo. For more details, we refer to McElreath [45] and Team [46].

Based on the results from the main analysis, one might get the impression that the number of levels for a group-level effect might be more important than the kind of grouping. In a supplementary analysis, however, we show that the WAIC value does not necessarily improve for a group-level effect with a larger number of levels. As depicted in Fig. 6, group-levels with more levels — "sentence" with 2,963 levels — do not per se obtain a better WAIC score than group-levels with fewer levels (here all other competing models).

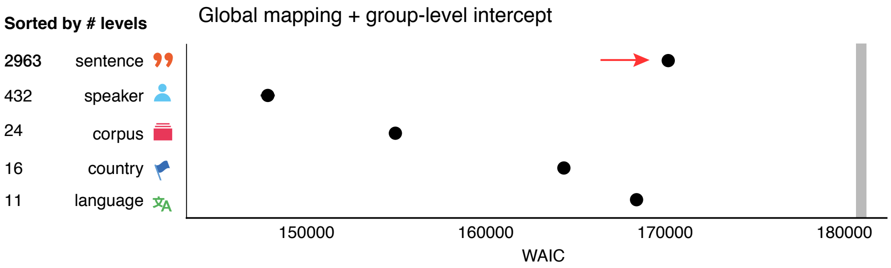

Supplementary Figure 6 | WAIC does not improve by a higher amount of levels in a group-level effect. The moderator "sentence" has by far the most levels, but it is the worst model compared to all other models that have one group-level effect with many fewer levels. In gray the performance of the base model.

ID	feature	subgroup	RC1	RC2	RC3	RC4	RC5	RC6	RC7
A1	F0semitoneFrom27.5Hz_sma3nz _amean	F0 percentiles	0.52	0.11	0.63	0.19	-0.02	0.13	-0.45
A2	F0semitoneFrom27.5Hz_sma3nz _stddevNorm	F0 variability	-0.11	-0.10	0.11	0.05	0.78	0.06	0.10
A3	F0semitoneFrom27.5Hz_sma3nz _percentile20.0	F0 percentiles	0.50	0.13	0.62	0.18	-0.16	0.12	-0.43
A4	F0semitoneFrom27.5Hz_sma3nz _percentile50.0	F0 percentiles	0.50	0.13	0.63	0.20	-0.02	0.12	-0.46
A5	F0semitoneFrom27.5Hz_sma3nz _percentile80.0	F0 percentiles	0.50	0.11	0.62	0.19	0.09	0.14	-0.46
A6	F0semitoneFrom27.5Hz_sma3nz _pctlrange0.2	F0 percentiles	0.13	-0.01	0.18	0.07	0.62	0.07	-0.18
B7	F0semitoneFrom27.5Hz_sma3nz _meanRisingSlope	F0 variability	0.06	-0.08	0.04	0.03	0.44	0.06	0.15
B8	F0semitoneFrom27.5Hz_sma3nz _stddevRisingSlope	F0 variability	0.04	-0.05	0.01	0.07	0.40	0.10	0.19
B9	F0semitoneFrom27.5Hz_sma3nz _meanFallingSlope	F0 variability	0.10	0.02	0.02	0.00	0.54	-0.04	-0.03

ID	feature	subgroup	RC1	RC2	RC3	RC4	RC5	RC6	RC7
B10	F0semitoneFrom27.5Hz_sma3nz _stddevFallingSlope	F0 variability	0.08	0.03	0.01	0.01	0.49	-0.01	-0.03
C11	loudness_sma3_amean	loudness	0.15	0.43	0.09	0.85	-0.03	-0.01	-0.04
C12	loudness_sma3_stddevNorm	loudness	0.19	-0.78	0.23	0.21	0.24	0.17	0.07
C13	loudness_sma3_percentile20.0	loudness	0.16	0.70	0.07	0.27	-0.12	-0.15	0.03
C14	loudness_sma3_percentile50.0	loudness	0.10	0.65	0.07	0.61	-0.07	-0.07	0.02
C15	loudness_sma3_percentile80.0	loudness	0.13	0.27	0.08	0.91	-0.01	0.03	-0.07
C16	loudness_sma3_pctlrange0.2	loudness	0.10	0.09	0.06	0.92	0.02	0.08	-0.09
C17	loudness_sma3_meanRisingSlope	loudness	0.16	0.11	0.09	0.89	0.09	0.07	-0.02
C18	loudness_sma3_stddevRisingSlope	loudness	0.14	-0.16	0.09	0.83	0.12	0.08	0.01
C19	loudness_sma3_meanFallingSlope	loudness	0.13	0.05	0.07	0.89	0.10	0.07	-0.01
C20	loudness_sma3_stddevFallingSlope	loudness	0.12	-0.08	0.07	0.83	0.13	0.07	0.01
D21	spectralFlux_sma3_amean	spectral flux	0.03	0.29	0.08	0.89	-0.00	-0.11	-0.06
D22	spectralFlux_sma3_stddevNorm	spectral flux	0.28	-0.76	0.17	0.12	0.22	0.23	0.01
E23	mfcc1_sma3_amean	MFCC1,2,4	-0.68	0.43	-0.05	-0.14	-0.23	-0.12	0.06
E24	mfcc1_sma3_stddevNorm	MFCC1,2,4	0.00	-0.01	0.00	-0.00	-0.01	0.01	-0.01
E25	mfcc2_sma3_amean	MFCC1,2,4	-0.82	-0.08	-0.02	-0.30	-0.04	-0.16	0.10
E26	mfcc2_sma3_stddevNorm	MFCC1,2,4	-0.01	0.01	-0.00	0.01	0.00	0.00	-0.01
E27	mfcc3_sma3_amean	MFCC3	-0.20	-0.01	0.01	0.00	0.05	-0.16	0.77
E28	mfcc3_sma3_stddevNorm	MFCC3	-0.00	0.01	-0.01	0.00	0.01	0.00	0.00
E29	mfcc4_sma3_amean	MFCC1,2,4	-0.79	-0.36	-0.10	-0.01	0.12	-0.00	0.12
E30	mfcc4_sma3_stddevNorm	MFCC1,2,4	-0.00	-0.00	-0.00	-0.01	0.01	0.00	0.01
G31	jitterLocal_sma3nz_amean	jitter	-0.06	0.00	0.19	0.10	0.58	-0.14	0.06
G32	jitterLocal_sma3nz_stddevNorm	jitter	0.04	0.04	0.34	0.05	0.13	0.43	-0.08
H33	shimmerLocaldB_sma3nz_amean	shimmer	-0.14	-0.11	0.29	0.02	0.44	-0.48	0.25
H34	shimmerLocaldB_sma3nz _stdde-vNorm	shimmer	-0.01	-0.01	0.43	0.22	-0.09	0.54	0.11
I35	HNRdBACF_sma3nz_amean	HNR	0.23	0.10	0.50	0.04	-0.33	0.48	-0.27
I36	HNRdBACF_sma3nz_stddevNorm	HNR	0.01	-0.01	0.01	0.02	0.11	-0.04	0.01
J37	logRelF0.H1.H2_sma3nz_amean	harmonic difference	-0.00	0.09	0.26	0.01	0.03	0.26	-0.01
J38	logRelF0.H1.H2_sma3nz_stddevNorm	harmonic difference	-0.01	-0.00	-0.00	0.01	-0.00	0.00	0.00
J39	logRelF0.H1.A3_sma3nz_amean	harmonic difference	-0.75	-0.02	0.37	-0.24	-0.07	0.03	0.15
J40	logRelF0.H1.A3_sma3nz_stddevNorm	harmonic difference	0.02	-0.01	-0.00	-0.00	0.03	-0.00	0.00
K41	F1frequency_sma3nz_amean	formants	0.53	-0.04	0.65	0.21	0.05	-0.02	-0.29
K42	F1frequency_sma3nz_stddevNorm	formants	-0.19	0.05	0.30	0.05	0.39	0.28	0.28
K43	F1bandwidth_sma3nz_amean	formants	-0.17	0.05	0.79	0.09	0.11	0.04	0.03
K44	F1bandwidth_sma3nz_stddevNorm	formants	0.42	0.00	0.39	0.13	0.32	0.15	0.08
K45	F1amplitudeLogRelF0_sma3nz _amean	formants	0.08	0.79	0.21	0.17	-0.08	0.47	-0.02
K46	F1amplitudeLogRelF0_sma3nz _stddevNorm	formants	-0.02	-0.28	-0.06	-0.04	0.02	-0.21	-0.00
K47	F2frequency_sma3nz_amean	formants	0.23	-0.06	0.88	0.18	0.10	0.05	-0.03
K48	F2frequency_sma3nz_stddevNorm	formants	-0.28	0.06	0.50	-0.13	0.18	0.30	0.19
K49	F2bandwidth_sma3nz_amean	formants	-0.09	-0.01	0.73	0.13	0.18	-0.04	-0.09
K50	F2bandwidth_sma3nz_stddevNorm	formants	0.20	0.04	0.44	0.03	0.13	0.18	0.35
K51	F2amplitudeLogRelF0_sma3nz _amean	formants	0.15	0.77	0.22	0.20	-0.06	0.47	-0.09
K52	F2amplitudeLogRelF0_sma3nz _stddevNorm	formants	-0.19	-0.71	-0.22	-0.21	0.05	-0.48	0.12
K53	F3frequency_sma3nz_amean	formants	0.07	0.00	0.94	0.12	0.09	0.07	-0.01
K54	F3frequency_sma3nz_stddevNorm	formants	-0.27	-0.09	0.47	-0.10	0.14	0.44	-0.01
K55	F3bandwidth_sma3nz_amean	formants	0.02	-0.14	0.76	0.16	0.18	0.02	0.06
K56	F3bandwidth_sma3nz_stddevNorm	formants	-0.11	0.10	0.45	0.01	0.06	0.23	0.13
K57	F3amplitudeLogRelF0_sma3nz _amean	formants	0.17	0.77	0.22	0.20	-0.06	0.47	-0.07
K58	F3amplitudeLogRelF0_sma3nz _stddevNorm	formants	-0.23	-0.69	-0.23	-0.24	0.02	-0.46	0.07
L59	alphaRatioV_sma3nz_amean	alpha ratio	0.72	0.02	-0.38	0.23	0.11	-0.05	-0.26
L60	alphaRatioV_sma3nz_stddevNorm	alpha ratio	-0.03	0.01	0.00	-0.01	-0.02	-0.01	0.02
M61	hammarbergIndexV_sma3nz_amean	Hammarberg index	-0.73	-0.07	0.47	-0.21	-0.09	0.11	0.07

ID	feature	subgroup	RC1	RC2	RC3	RC4	RC5	RC6	RC7
M62	hammarbergIndexV_sma3nz _std-devNorm	Hammarberg index	0.42	-0.12	0.10	0.14	0.28	0.14	0.02
N63	slopeV0.500_sma3nz_amean	spectral slope	0.83	0.09	0.22	-0.03	0.01	0.11	-0.07
N64	slopeV0.500_sma3nz_stddevNorm	spectral slope	-0.00	0.00	0.00	-0.00	0.01	-0.01	-0.01
N65	slopeV500.1500_sma3nz_amean	spectral slope	0.23	-0.15	-0.37	0.00	0.29	0.29	-0.14
N66	slopeV500.1500_sma3nz_stddevNorm	spectral slope	0.00	0.00	-0.00	0.01	-0.01	-0.01	0.01
D67	spectralFluxV_sma3nz_amean	spectral flux	-0.00	-0.07	0.08	0.92	0.03	-0.10	-0.11
D68	spectralFluxV_sma3nz_stddevNorm	spectral flux	0.30	-0.13	0.38	0.10	0.25	0.49	-0.06
E69	mfcc1V_sma3nz_amean	MFCC1,2,4	-0.55	-0.10	0.40	-0.23	-0.15	0.06	0.41
E70	mfcc1V_sma3nz_stddevNorm	MFCC1,2,4	0.01	0.01	-0.00	0.02	0.01	0.01	-0.01
E71	mfcc2V_sma3nz_amean	MFCC1,2,4	-0.83	0.05	0.04	-0.24	-0.01	-0.02	0.24
E72	mfcc2V_sma3nz_stddevNorm	MFCC1,2,4	0.01	0.00	0.00	-0.00	0.00	0.01	0.00
F73	mfcc3V_sma3nz_amean	MFCC3	0.06	-0.13	0.03	-0.00	0.13	-0.03	0.89
F74	mfcc3V_sma3nz_stddevNorm	MFCC3	-0.00	0.01	-0.00	-0.00	0.00	0.02	0.00
E75	mfcc4V_sma3nz_amean	MFCC1,2,4	-0.78	-0.11	-0.21	0.02	0.12	0.07	0.20
E76	mfcc4V_sma3nz_stddevNorm	MFCC1,2,4	-0.01	-0.00	0.00	-0.01	0.01	-0.01	-0.00
L77	alphaRatioUV_sma3nz_amean	alpha ratio	0.74	-0.18	0.09	0.02	0.09	0.26	0.22
M78	hammarbergIndexUV_sma3nz _amean	Hammarberg index	-0.76	0.13	-0.12	0.01	-0.07	-0.21	-0.26
N79	slopeUV0.500_sma3nz_amean	spectral slope	0.81	0.01	-0.01	-0.06	0.05	-0.02	0.28
N80	slopeUV500.1500_sma3nz_amean	spectral slope	0.20	-0.61	-0.00	-0.05	0.13	0.53	0.13
D81	spectralFluxUV_sma3nz_amean	spectral flux	0.02	0.44	0.08	0.58	0.04	-0.30	0.10
O82	loudnessPeaksPerSec	duration	0.05	0.74	-0.29	0.05	-0.07	-0.12	0.04
O83	VoicedSegmentsPerSec	duration	-0.02	0.59	0.23	0.11	0.30	-0.28	0.09
O84	MeanVoicedSegmentLengthSec	duration	0.06	0.12	0.13	0.01	-0.29	0.69	-0.14
O85	StddevVoicedSegmentLengthSec	duration	0.01	0.07	0.12	0.11	-0.15	0.61	-0.05
O86	MeanUnvoicedSegmentLength	duration	0.01	-0.76	0.02	-0.12	-0.12	-0.15	-0.02
O87	StddevUnvoicedSegmentLength	duration	0.04	-0.82	0.12	0.04	0.04	-0.09	0.10
O88	equivalentSoundLevel_dBp	duration	-0.02	0.12	0.39	0.80	0.08	0.16	0.07

Supplementary Table 5 | Raw loading displayed in Supplementary Figure 4 and used in Figure 1d.

Factor	Global Anger	Kenyan Anger	Male Anger	Speaker CK Anger	Combined Anger
RC1	1.31	-1.79	3.71	-1.18	2.05
RC2	1.63	0.69	3.87	0.77	6.95
RC3	0.54	0.98	1.11	-0.34	2.29
RC4	-0.34	-0.47	-0.05	0.04	-0.83
RC5	0.58	0.72	0.06	-0.23	1.13
RC6	1.17	0.80	0.20	0.06	2.23
RC7	-0.64	1.33	-0.49	0.63	0.82

Supplementary Table 6 | Mean estimates displayed in Figure 1d.

Factor	Anger	Disgust	Fear	Happiness	Sadness	Surprise
RC1	1.92 [1.30, 2.52]	0.36 [-0.27, 1.01]	0.87 [0.20, 1.53]	1.20 [0.72, 1.66]	-0.49 [-0.96, 0.01]	0.67 [-0.02, 1.36]
RC2	2.65 [1.94, 3.33]	0.26 [-0.14, 0.66]	0.91 [0.30, 1.50]	1.76 [1.09, 2.40]	-1.24 [-1.87, -0.56]	0.93 [0.31, 1.53]
RC3	0.21 [-0.35, 0.77]	-0.14 [-0.62, 0.34]	0.83 [0.16, 1.46]	0.69 [0.10, 1.27]	0.71 [0.15, 1.25]	1.37 [0.62, 2.12]
RC4	-0.39 [-0.76, -0.03]	-0.81 [-1.39, -0.22]	-0.39 [-0.94, 0.14]	-0.05 [-0.39, 0.30]	-1.55 [-2.12, -0.98]	-0.15 [-0.75, 0.44]
RC5	0.71 [0.26, 1.16]	0.26 [-0.12, 0.64]	-0.33 [-0.79, 0.13]	0.66 [0.27, 1.04]	0.34 [0.00, 0.69]	-0.03 [-0.59, 0.54]
RC6	1.18 [0.84, 1.52]	0.61 [0.15, 1.05]	0.01 [-0.28, 0.30]	0.85 [0.50, 1.19]	0.20 [-0.09, 0.49]	1.06 [0.45, 1.62]
RC7	-0.76 [-1.24, -0.26]	-0.67 [-1.01, -0.33]	-1.14 [-1.75, -0.52]	-1.31 [-1.72, -0.89]	-0.17 [-0.48, 0.15]	-1.67 [-2.29, -1.00]

Supplementary Table 7 | Mean estimates with credible interval displayed in Figure 2a.

Factor	Global Anger	Corpus SAV Anger	Combined Anger
RC1	1.92	0.89	2.81
RC2	2.65	-0.19	2.47
RC3	0.21	0.15	0.36
RC4	-0.39	0.65	0.26
RC5	0.71	1.96	2.68
RC6	1.18	0.27	1.45
RC7	-0.76	-0.24	-1.00

Supplementary Table 8 | Mean estimates displayed in Figure 2b.

Emotion	Factor	Corpus	Mean and CI
Anger	RC1	ESC	4.10 [3.45, 4.80]
Anger	RC1	DBA	1.79 [0.99, 2.66]
Anger	RC1	VEN	1.75 [1.06, 2.46]
Anger	RC1	ENT	0.97 [0.11, 1.84]
Anger	RC1	CFE	2.52 [2.00, 3.05]
Anger	RC1	PAX	4.44 [3.86, 5.03]
Anger	RC1	RAV	1.14 [0.70, 1.59]
Anger	RC1	TES	3.07 [2.41, 3.77]
Anger	RC1	EEK	0.61 [0.03, 1.20]
Anger	RC1	EDB	4.95 [4.14, 5.78]
Anger	RC1	SEH	2.86 [2.64, 3.08]
Anger	RC1	SES	0.83 [0.72, 0.94]
Anger	RC1	DAF	0.65 [-0.55, 1.84]
Anger	RC1	EHI	3.65 [2.30, 5.11]
Anger	RC1	HAW	3.35 [1.47, 5.32]
Anger	RC1	AH1	0.99 [0.16, 1.82]
Anger	RC1	AH2	4.61 [4.32, 4.90]
Anger	RC1	J01	1.98 [0.89, 3.07]
Anger	RC1	SAV	2.81 [1.77, 3.89]
Anger	RC1	CRE	0.32 [0.08, 0.56]
Anger	RC1	VDB	4.62 [4.22, 5.03]
Anger	RC1	IMP	0.44 [0.17, 0.71]
Anger	RC1	EMA	-1.26 [-2.88, 0.29]
Anger	RC2	ESC	7.26 [6.41, 8.19]
Anger	RC2	DBA	2.57 [1.73, 3.45]
Anger	RC2	VEN	2.48 [1.93, 3.05]
Anger	RC2	ENT	2.37 [1.65, 3.09]
Anger	RC2	CFE	4.41 [3.19, 5.65]
Anger	RC2	PAX	4.40 [3.63, 5.18]
Anger	RC2	RAV	5.25 [4.57, 5.96]
Anger	RC2	TES	5.35 [4.27, 6.50]
Anger	RC2	EEK	1.12 [0.78, 1.46]
Anger	RC2	EDB	2.58 [1.77, 3.40]
Anger	RC2	SEH	2.11 [1.91, 2.32]
Anger	RC2	SES	1.74 [1.62, 1.86]
Anger	RC2	DAF	3.63 [1.40, 5.82]
Anger	RC2	EHI	3.11 [1.38, 4.99]
Anger	RC2	HAW	2.14 [1.26, 3.05]
Anger	RC2	AH1	0.54 [0.23, 0.85]
Anger	RC2	AH2	3.83 [3.53, 4.13]
Anger	RC2	J01	2.76 [1.86, 3.67]
Anger	RC2	SAV	2.47 [2.06, 2.90]
Anger	RC2	CRE	3.81 [3.55, 4.07]
Anger	RC2	VDB	-0.15 [-0.52, 0.22]
Anger	RC2	IMP	0.98 [0.83, 1.13]
Anger	RC2	EMA	10.75 [9.05, 12.61]
Anger	RC3	ESC	2.51 [1.47, 3.60]
Anger	RC3	DBA	0.70 [-0.54, 1.97]

Emotion	Factor	Corpus	Mean and CI
Anger	RC3	VEN	1.00 [0.11, 1.99]
Anger	RC3	ENT	0.69 [-0.15, 1.53]
Anger	RC3	CFE	0.31 [-0.49, 1.13]
Anger	RC3	PAX	2.98 [2.33, 3.65]
Anger	RC3	RAV	1.68 [1.00, 2.38]
Anger	RC3	TES	3.05 [2.03, 4.10]
Anger	RC3	EEK	0.35 [-0.29, 0.96]
Anger	RC3	EDB	1.46 [0.25, 2.72]
Anger	RC3	SEH	-1.68 [-2.08, -1.28]
Anger	RC3	SES	-0.37 [-0.42, -0.33]
Anger	RC3	DAF	0.62 [-0.71, 1.93]
Anger	RC3	EHI	-1.09 [-2.55, 0.44]
Anger	RC3	HAW	-1.65 [-3.62, 0.19]
Anger	RC3	AH1	-3.56 [-4.67, -2.47]
Anger	RC3	AH2	-1.66 [-1.94, -1.38]
Anger	RC3	J01	-0.43 [-1.50, 0.68]
Anger	RC3	SAV	0.36 [-0.76, 1.49]
Anger	RC3	CRE	0.97 [0.76, 1.17]
Anger	RC3	VDB	0.06 [-0.43, 0.56]
Anger	RC3	IMP	1.44 [1.07, 1.83]
Anger	RC3	EMA	2.17 [0.68, 3.67]
Anger	RC4	ESC	-0.32 [-1.03, 0.38]
Anger	RC4	DBA	0.04 [-0.88, 1.02]
Anger	RC4	VEN	-1.19 [-1.91, -0.53]
Anger	RC4	ENT	0.30 [-0.32, 0.94]
Anger	RC4	CFE	-1.30 [-1.97, -0.65]
Anger	RC4	PAX	-1.15 [-1.75, -0.54]
Anger	RC4	RAV	-0.42 [-1.18, 0.34]
Anger	RC4	TES	0.23 [-0.76, 1.22]
Anger	RC4	EEK	-0.28 [-0.67, 0.12]
Anger	RC4	EDB	-1.17 [-2.00, -0.35]
Anger	RC4	SEH	-0.45 [-0.59, -0.29]
Anger	RC4	SES	0.31 [0.17, 0.44]
Anger	RC4	DAF	-1.77 [-2.54, -1.03]
Anger	RC4	EHI	0.27 [-0.59, 1.18]
Anger	RC4	HAW	-0.62 [-2.05, 0.74]
Anger	RC4	AH1	-0.83 [-1.14, -0.52]
Anger	RC4	AH2	0.61 [0.47, 0.76]
Anger	RC4	J01	0.63 [-0.23, 1.52]
Anger	RC4	SAV	0.26 [-0.66, 1.15]
Anger	RC4	CRE	-0.23 [-0.44, -0.01]
Anger	RC4	VDB	-0.13 [-0.40, 0.15]
Anger	RC4	IMP	-0.22 [-0.38, -0.06]
Anger	RC4	EMA	-3.12 [-4.24, -2.02]
Anger	RC5	ESC	1.13 [0.57, 1.71]
Anger	RC5	DBA	1.08 [0.34, 1.83]
Anger	RC5	VEN	0.82 [0.27, 1.37]
Anger	RC5	ENT	0.11 [-0.44, 0.65]
Anger	RC5	CFE	0.86 [0.42, 1.29]
Anger	RC5	PAX	3.33 [2.95, 3.72]
Anger	RC5	RAV	0.37 [-0.11, 0.88]
Anger	RC5	TES	2.71 [2.19, 3.23]
Anger	RC5	EEK	0.26 [0.00, 0.53]
Anger	RC5	EDB	1.24 [0.59, 1.89]
Anger	RC5	SEH	0.57 [0.41, 0.73]
Anger	RC5	SES	-0.36 [-0.48, -0.25]
Anger	RC5	DAF	-1.36 [-2.28, -0.46]
Anger	RC5	EHI	1.17 [0.23, 2.16]
Anger	RC5	HAW	0.96 [-0.35, 2.27]
Anger	RC5	AH1	-0.58 [-1.07, -0.11]

Emotion	Factor	Corpus	Mean and CI
Anger	RC5	AH2	0.15 [-0.05, 0.35]
Anger	RC5	J01	-0.40 [-1.13, 0.32]
Anger	RC5	SAV	2.68 [1.98, 3.42]
Anger	RC5	CRE	0.16 [0.00, 0.32]
Anger	RC5	VDB	3.82 [3.50, 4.16]
Anger	RC5	IMP	1.05 [0.78, 1.32]
Anger	RC5	EMA	0.30 [-0.71, 1.28]
Anger	RC6	ESC	0.90 [0.46, 1.35]
Anger	RC6	DBA	1.91 [1.28, 2.60]
Anger	RC6	VEN	1.42 [0.94, 1.93]
Anger	RC6	ENT	0.39 [-0.06, 0.85]
Anger	RC6	CFE	1.54 [1.19, 1.90]
Anger	RC6	PAX	2.50 [2.17, 2.83]
Anger	RC6	RAV	1.42 [1.07, 1.78]
Anger	RC6	TES	4.25 [3.76, 4.74]
Anger	RC6	EEK	0.64 [0.43, 0.86]
Anger	RC6	EDB	1.48 [1.07, 1.92]
Anger	RC6	SEH	0.46 [0.35, 0.57]
Anger	RC6	SES	0.20 [0.12, 0.30]
Anger	RC6	DAF	1.93 [1.40, 2.48]
Anger	RC6	EHI	1.44 [0.70, 2.23]
Anger	RC6	HAW	1.42 [0.57, 2.34]
Anger	RC6	AH1	0.88 [0.46, 1.31]
Anger	RC6	AH2	1.62 [1.47, 1.78]
Anger	RC6	J01	1.28 [0.75, 1.82]
Anger	RC6	SAV	1.45 [1.00, 1.91]
Anger	RC6	CRE	0.64 [0.56, 0.73]
Anger	RC6	VDB	0.79 [0.59, 0.98]
Anger	RC6	IMP	0.49 [0.34, 0.65]
Anger	RC6	EMA	2.70 [1.99, 3.46]
Anger	RC7	ESC	-0.81 [-1.17, -0.47]
Anger	RC7	DBA	-1.09 [-1.78, -0.42]
Anger	RC7	VEN	-0.01 [-0.47, 0.45]
Anger	RC7	ENT	0.14 [-0.42, 0.69]
Anger	RC7	CFE	-0.93 [-1.26, -0.60]
Anger	RC7	PAX	-2.61 [-2.97, -2.24]
Anger	RC7	RAV	0.31 [-0.04, 0.65]
Anger	RC7	TES	-5.59 [-6.28, -4.91]
Anger	RC7	EEK	0.19 [-0.17, 0.55]
Anger	RC7	EDB	-1.44 [-1.94, -0.96]
Anger	RC7	SEH	-1.46 [-1.61, -1.31]
Anger	RC7	SES	-0.78 [-0.88, -0.69]
Anger	RC7	DAF	-2.04 [-2.75, -1.33]
Anger	RC7	EHI	-0.32 [-1.15, 0.55]
Anger	RC7	HAW	-0.77 [-2.02, 0.50]
Anger	RC7	AH1	-1.48 [-1.97, -0.99]
Anger	RC7	AH2	0.26 [0.07, 0.46]
Anger	RC7	J01	-0.51 [-1.17, 0.15]
Anger	RC7	SAV	-1.00 [-1.66, -0.32]
Anger	RC7	CRE	-0.07 [-0.20, 0.06]
Anger	RC7	VDB	-2.05 [-2.30, -1.80]
Anger	RC7	IMP	0.03 [-0.18, 0.25]
Anger	RC7	EMA	1.92 [1.23, 2.65]
Happiness	RC1	DBA	1.43 [0.73, 2.18]
Happiness	RC1	VEN	0.89 [0.29, 1.50]
Happiness	RC1	ENT	1.14 [0.29, 1.99]
Happiness	RC1	CFE	1.59 [1.11, 2.11]
Happiness	RC1	PAX	1.51 [1.03, 1.99]
Happiness	RC1	RAV	0.90 [0.50, 1.31]
Happiness	RC1	TES	3.04 [2.36, 3.75]

Emotion	Factor	Corpus	Mean and CI
Happiness	RC1	EEK	3.26 [2.61, 3.91]
Happiness	RC1	EDB	2.81 [2.08, 3.56]
Happiness	RC1	SEH	1.17 [0.99, 1.35]
Happiness	RC1	SES	0.09 [-0.01, 0.20]
Happiness	RC1	DAF	1.47 [0.38, 2.58]
Happiness	RC1	EHI	1.07 [-0.13, 2.24]
Happiness	RC1	HAW	1.73 [0.16, 3.25]
Happiness	RC1	AH1	0.71 [-0.05, 1.51]
Happiness	RC1	AH2	2.76 [2.47, 3.05]
Happiness	RC1	J01	1.03 [0.04, 2.02]
Happiness	RC1	SAV	3.58 [2.60, 4.58]
Happiness	RC1	CRE	-0.40 [-0.61, -0.18]
Happiness	RC1	IMP	0.52 [0.26, 0.78]
Happiness	RC1	EMA	-1.19 [-2.53, 0.13]
Happiness	RC2	DBA	1.25 [0.53, 2.00]
Happiness	RC2	VEN	1.69 [1.20, 2.21]
Happiness	RC2	ENT	1.28 [0.56, 1.99]
Happiness	RC2	CFE	1.81 [0.80, 2.85]
Happiness	RC2	PAX	3.23 [2.59, 3.89]
Happiness	RC2	RAV	3.45 [2.79, 4.12]
Happiness	RC2	TES	7.70 [6.62, 8.85]
Happiness	RC2	EEK	-0.48 [-0.99, 0.00]
Happiness	RC2	EDB	1.77 [0.97, 2.60]
Happiness	RC2	SEH	1.08 [0.89, 1.27]
Happiness	RC2	SES	-0.22 [-0.34, -0.10]
Happiness	RC2	DAF	3.08 [1.25, 4.86]
Happiness	RC2	EHI	1.83 [0.15, 3.51]
Happiness	RC2	HAW	1.89 [1.10, 2.78]
Happiness	RC2	AH1	0.98 [0.69, 1.29]
Happiness	RC2	AH2	4.30 [3.99, 4.61]
Happiness	RC2	J01	1.88 [0.99, 2.76]
Happiness	RC2	SAV	1.96 [1.56, 2.36]
Happiness	RC2	CRE	2.60 [2.35, 2.86]
Happiness	RC2	IMP	0.80 [0.66, 0.96]
Happiness	RC2	EMA	8.52 [6.91, 10.32]
Happiness	RC3	DBA	0.13 [-0.85, 1.11]
Happiness	RC3	VEN	-0.43 [-1.26, 0.40]
Happiness	RC3	ENT	-0.15 [-0.97, 0.66]
Happiness	RC3	CFE	0.09 [-0.59, 0.78]
Happiness	RC3	PAX	0.73 [0.23, 1.21]
Happiness	RC3	RAV	0.48 [-0.11, 1.08]
Happiness	RC3	TES	9.10 [8.02, 10.19]
Happiness	RC3	EEK	1.40 [0.68, 2.11]
Happiness	RC3	EDB	1.74 [0.52, 2.91]
Happiness	RC3	SEH	0.36 [0.05, 0.66]
Happiness	RC3	SES	0.05 [0.01, 0.08]
Happiness	RC3	DAF	0.87 [-0.46, 2.17]
Happiness	RC3	EHI	2.73 [1.10, 4.54]
Happiness	RC3	HAW	2.08 [0.22, 4.01]
Happiness	RC3	AH1	0.47 [-0.52, 1.48]
Happiness	RC3	AH2	-0.03 [-0.31, 0.26]
Happiness	RC3	J01	-0.53 [-1.61, 0.52]
Happiness	RC3	SAV	2.07 [1.06, 3.07]
Happiness	RC3	CRE	0.46 [0.32, 0.61]
Happiness	RC3	IMP	0.50 [0.14, 0.87]
Happiness	RC3	EMA	3.21 [1.85, 4.59]
Happiness	RC4	DBA	0.22 [-0.58, 0.98]
Happiness	RC4	VEN	-0.02 [-0.71, 0.64]
Happiness	RC4	ENT	0.16 [-0.45, 0.77]
Happiness	RC4	CFE	0.41 [-0.17, 1.03]

Emotion	Factor	Corpus	Mean and CI
Happiness	RC4	PAX	0.27 [-0.23, 0.76]
Happiness	RC4	RAV	0.36 [-0.35, 1.09]
Happiness	RC4	TES	-1.77 [-2.78, -0.80]
Happiness	RC4	EEK	-0.20 [-0.63, 0.21]
Happiness	RC4	EDB	-0.25 [-1.01, 0.52]
Happiness	RC4	SEH	0.42 [0.28, 0.55]
Happiness	RC4	SES	-0.01 [-0.13, 0.12]
Happiness	RC4	DAF	-2.12 [-3.00, -1.26]
Happiness	RC4	EHI	0.06 [-0.72, 0.84]
Happiness	RC4	HAW	-0.08 [-1.38, 1.22]
Happiness	RC4	AH1	0.00 [-0.28, 0.30]
Happiness	RC4	AH2	-0.09 [-0.24, 0.06]
Happiness	RC4	J01	0.82 [0.05, 1.61]
Happiness	RC4	SAV	1.38 [0.51, 2.29]
Happiness	RC4	CRE	-0.17 [-0.37, 0.03]
Happiness	RC4	IMP	0.19 [0.03, 0.35]
Happiness	RC4	EMA	-1.08 [-2.11, -0.11]
Happiness	RC5	DBA	0.23 [-0.39, 0.86]
Happiness	RC5	VEN	0.99 [0.48, 1.50]
Happiness	RC5	ENT	-0.15 [-0.69, 0.39]
Happiness	RC5	CFE	0.78 [0.37, 1.19]
Happiness	RC5	PAX	1.89 [1.58, 2.21]
Happiness	RC5	RAV	1.26 [0.81, 1.73]
Happiness	RC5	TES	3.98 [3.45, 4.51]
Happiness	RC5	EEK	0.96 [0.66, 1.25]
Happiness	RC5	EDB	1.78 [1.11, 2.45]
Happiness	RC5	SEH	0.64 [0.50, 0.78]
Happiness	RC5	SES	-0.23 [-0.33, -0.13]
Happiness	RC5	DAF	0.33 [-0.51, 1.16]
Happiness	RC5	EHI	0.94 [-0.05, 1.96]
Happiness	RC5	HAW	2.10 [0.79, 3.57]
Happiness	RC5	AH1	-0.19 [-0.67, 0.28]
Happiness	RC5	AH2	-0.05 [-0.26, 0.15]
Happiness	RC5	J01	-0.07 [-0.75, 0.63]
Happiness	RC5	SAV	2.06 [1.41, 2.74]
Happiness	RC5	CRE	-0.24 [-0.38, -0.10]
Happiness	RC5	IMP	0.78 [0.52, 1.04]
Happiness	RC5	EMA	1.51 [0.62, 2.43]
Happiness	RC6	DBA	0.99 [0.51, 1.52]
Happiness	RC6	VEN	0.67 [0.25, 1.10]
Happiness	RC6	ENT	0.50 [0.06, 0.95]
Happiness	RC6	CFE	0.54 [0.22, 0.89]
Happiness	RC6	PAX	1.28 [1.00, 1.56]
Happiness	RC6	RAV	1.03 [0.71, 1.36]
Happiness	RC6	TES	5.20 [4.70, 5.72]
Happiness	RC6	EEK	-0.22 [-0.48, 0.04]
Happiness	RC6	EDB	1.03 [0.63, 1.45]
Happiness	RC6	SEH	0.66 [0.57, 0.75]
Happiness	RC6	SES	-0.15 [-0.23, -0.07]
Happiness	RC6	DAF	1.25 [0.62, 1.86]
Happiness	RC6	EHI	1.60 [0.80, 2.47]
Happiness	RC6	HAW	1.07 [0.28, 1.87]
Happiness	RC6	AH1	0.75 [0.32, 1.18]
Happiness	RC6	AH2	2.30 [2.13, 2.46]
Happiness	RC6	J01	1.05 [0.56, 1.55]
Happiness	RC6	SAV	1.43 [1.00, 1.87]
Happiness	RC6	CRE	0.50 [0.43, 0.58]
Happiness	RC6	IMP	0.42 [0.27, 0.57]
Happiness	RC6	EMA	3.57 [2.89, 4.27]
Happiness	RC7	DBA	-1.25 [-1.82, -0.68]

Emotion	Factor	Corpus	Mean and CI
Happiness	RC7	VEN	-0.85 [-1.29, -0.44]
Happiness	RC7	ENT	-0.37 [-0.90, 0.17]
Happiness	RC7	CFE	-0.94 [-1.27, -0.62]
Happiness	RC7	PAX	-1.70 [-2.03, -1.39]
Happiness	RC7	RAV	-0.36 [-0.68, -0.03]
Happiness	RC7	TES	-6.13 [-6.83, -5.43]
Happiness	RC7	EEK	-1.47 [-1.87, -1.06]
Happiness	RC7	EDB	-2.25 [-2.76, -1.76]
Happiness	RC7	SEH	-1.34 [-1.48, -1.20]
Happiness	RC7	SES	-0.27 [-0.35, -0.18]
Happiness	RC7	DAF	-1.41 [-2.13, -0.73]
Happiness	RC7	EHI	-2.91 [-3.93, -1.94]
Happiness	RC7	HAW	-2.55 [-3.82, -1.38]
Happiness	RC7	AH1	-2.27 [-2.77, -1.80]
Happiness	RC7	AH2	-1.78 [-1.99, -1.57]
Happiness	RC7	J01	-1.56 [-2.21, -0.94]
Happiness	RC7	SAV	-2.97 [-3.66, -2.32]
Happiness	RC7	CRE	-0.87 [-0.99, -0.75]
Happiness	RC7	IMP	-0.31 [-0.52, -0.09]
Happiness	RC7	EMA	0.31 [-0.30, 0.95]
Sadness	RC1	DBA	0.38 [-0.33, 1.09]
Sadness	RC1	VEN	-0.25 [-0.81, 0.30]
Sadness	RC1	ENT	-0.05 [-0.92, 0.83]
Sadness	RC1	CFE	1.05 [0.56, 1.54]
Sadness	RC1	PAX	-2.90 [-3.50, -2.31]
Sadness	RC1	RAV	-0.02 [-0.42, 0.39]
Sadness	RC1	TES	-0.82 [-1.50, -0.10]
Sadness	RC1	EEK	-0.73 [-1.35, -0.14]
Sadness	RC1	EDB	-3.71 [-4.99, -2.59]
Sadness	RC1	SEH	0.07 [-0.12, 0.24]
Sadness	RC1	DAF	-1.89 [-3.22, -0.63]
Sadness	RC1	EHI	-0.38 [-1.49, 0.76]
Sadness	RC1	HAW	0.44 [-0.92, 1.85]
Sadness	RC1	AH1	-2.44 [-3.54, -1.38]
Sadness	RC1	AH2	-0.40 [-0.68, -0.13]
Sadness	RC1	J01	-0.08 [-1.07, 0.91]
Sadness	RC1	GEM	-1.58 [-3.15, -0.11]
Sadness	RC1	SAV	0.23 [-0.56, 1.03]
Sadness	RC1	CRE	-1.16 [-1.39, -0.94]
Sadness	RC1	IMP	-0.52 [-0.80, -0.26]
Sadness	RC1	EMA	1.22 [-0.05, 2.57]
Sadness	RC2	DBA	-0.63 [-1.41, 0.14]
Sadness	RC2	VEN	0.25 [-0.27, 0.78]
Sadness	RC2	ENT	-1.81 [-2.54, -1.07]
Sadness	RC2	CFE	-1.40 [-2.66, -0.19]
Sadness	RC2	PAX	-3.38 [-4.43, -2.38]
Sadness	RC2	RAV	-0.30 [-1.03, 0.44]
Sadness	RC2	TES	-1.04 [-2.31, 0.25]
Sadness	RC2	EEK	-1.22 [-1.81, -0.67]
Sadness	RC2	EDB	0.20 [-0.89, 1.32]
Sadness	RC2	SEH	0.27 [0.08, 0.46]
Sadness	RC2	DAF	-0.35 [-2.37, 1.73]
Sadness	RC2	EHI	-1.42 [-3.50, 0.63]
Sadness	RC2	HAW	0.11 [-0.71, 0.91]
Sadness	RC2	AH1	-3.56 [-4.14, -3.00]
Sadness	RC2	AH2	-5.43 [-5.78, -5.10]
Sadness	RC2	J01	-0.17 [-1.20, 0.82]
Sadness	RC2	GEM	-3.76 [-6.00, -1.63]
Sadness	RC2	SAV	0.31 [-0.14, 0.76]
Sadness	RC2	CRE	-3.25 [-3.67, -2.85]

Emotion	Factor	Corpus	Mean and CI
Sadness	RC2	IMP	-0.54 [-0.73, -0.35]
Sadness	RC2	EMA	-3.84 [-5.07, -2.68]
Sadness	RC3	DBA	-0.51 [-1.49, 0.45]
Sadness	RC3	VEN	1.07 [0.30, 1.88]
Sadness	RC3	ENT	-0.42 [-1.22, 0.38]
Sadness	RC3	CFE	0.06 [-0.66, 0.78]
Sadness	RC3	PAX	2.68 [2.02, 3.35]
Sadness	RC3	RAV	1.00 [0.44, 1.58]
Sadness	RC3	TES	7.30 [6.34, 8.28]
Sadness	RC3	EEK	-0.02 [-0.69, 0.64]
Sadness	RC3	EDB	2.01 [0.74, 3.37]
Sadness	RC3	SEH	1.93 [1.63, 2.22]
Sadness	RC3	DAF	0.81 [-0.44, 2.03]
Sadness	RC3	EHI	-1.50 [-3.06, -0.11]
Sadness	RC3	HAW	1.22 [-0.61, 3.09]
Sadness	RC3	AH1	-0.93 [-2.19, 0.32]
Sadness	RC3	AH2	-0.10 [-0.35, 0.14]
Sadness	RC3	J01	1.46 [0.50, 2.45]
Sadness	RC3	GEM	-0.85 [-2.19, 0.34]
Sadness	RC3	SAV	1.88 [1.01, 2.77]
Sadness	RC3	CRE	-0.26 [-0.35, -0.17]
Sadness	RC3	IMP	0.51 [0.20, 0.82]
Sadness	RC3	EMA	1.88 [1.02, 2.74]
Sadness	RC4	DBA	-0.95 [-2.03, 0.15]
Sadness	RC4	VEN	-1.53 [-2.20, -0.87]
Sadness	RC4	ENT	-0.70 [-1.34, -0.06]
Sadness	RC4	CFE	-1.06 [-1.70, -0.41]
Sadness	RC4	PAX	-3.47 [-4.18, -2.77]
Sadness	RC4	RAV	-1.41 [-2.24, -0.59]
Sadness	RC4	TES	-5.75 [-6.75, -4.76]
Sadness	RC4	EEK	-1.15 [-1.59, -0.71]
Sadness	RC4	EDB	-5.62 [-7.12, -4.23]
Sadness	RC4	SEH	-0.22 [-0.36, -0.09]
Sadness	RC4	DAF	-3.51 [-4.32, -2.74]
Sadness	RC4	EHI	-1.24 [-2.36, -0.14]
Sadness	RC4	HAW	-1.86 [-3.73, -0.07]
Sadness	RC4	AH1	1.72 [1.22, 2.22]
Sadness	RC4	AH2	0.26 [0.11, 0.41]
Sadness	RC4	J01	-2.04 [-3.03, -1.06]
Sadness	RC4	GEM	-1.72 [-3.60, 0.07]
Sadness	RC4	SAV	-2.32 [-3.23, -1.38]
Sadness	RC4	CRE	-1.51 [-1.74, -1.29]
Sadness	RC4	IMP	-0.50 [-0.66, -0.34]
Sadness	RC4	EMA	-3.71 [-4.61, -2.85]
Sadness	RC5	DBA	0.63 [0.02, 1.24]
Sadness	RC5	VEN	0.73 [0.26, 1.21]
Sadness	RC5	ENT	0.33 [-0.21, 0.86]
Sadness	RC5	CFE	0.94 [0.55, 1.34]
Sadness	RC5	PAX	-0.21 [-0.60, 0.19]
Sadness	RC5	RAV	0.16 [-0.26, 0.58]
Sadness	RC5	TES	1.70 [1.22, 2.18]
Sadness	RC5	EEK	0.68 [0.40, 0.97]
Sadness	RC5	EDB	0.29 [-0.56, 1.11]
Sadness	RC5	SEH	-0.13 [-0.27, 0.02]
Sadness	RC5	DAF	0.22 [-0.51, 0.95]
Sadness	RC5	EHI	-0.06 [-0.87, 0.70]
Sadness	RC5	HAW	0.76 [-0.34, 1.84]
Sadness	RC5	AH1	-1.30 [-1.93, -0.68]
Sadness	RC5	AH2	-1.91 [-2.10, -1.71]
Sadness	RC5	J01	1.19 [0.52, 1.90]

Emotion	Factor	Corpus	Mean and CI
Sadness	RC5	GEM	-0.68 [-1.73, 0.37]
Sadness	RC5	SAV	1.73 [1.19, 2.28]
Sadness	RC5	CRE	-0.06 [-0.20, 0.08]
Sadness	RC5	IMP	0.24 [-0.01, 0.49]
Sadness	RC5	EMA	1.72 [1.02, 2.45]
Sadness	RC6	DBA	-0.46 [-1.00, 0.07]
Sadness	RC6	VEN	0.63 [0.21, 1.07]
Sadness	RC6	ENT	-0.22 [-0.67, 0.23]
Sadness	RC6	CFE	0.29 [-0.04, 0.63]
Sadness	RC6	PAX	1.42 [1.10, 1.73]
Sadness	RC6	RAV	0.76 [0.43, 1.10]
Sadness	RC6	TES	3.32 [2.90, 3.77]
Sadness	RC6	EEK	-0.14 [-0.40, 0.12]
Sadness	RC6	EDB	0.62 [0.10, 1.14]
Sadness	RC6	SEH	0.44 [0.35, 0.54]
Sadness	RC6	DAF	-0.06 [-0.70, 0.54]
Sadness	RC6	EHI	-1.12 [-1.90, -0.43]
Sadness	RC6	HAW	0.66 [-0.19, 1.50]
Sadness	RC6	AH1	-0.85 [-1.43, -0.26]
Sadness	RC6	AH2	0.23 [0.08, 0.38]
Sadness	RC6	J01	0.50 [0.01, 1.00]
Sadness	RC6	GEM	-0.40 [-1.34, 0.53]
Sadness	RC6	SAV	0.73 [0.27, 1.18]
Sadness	RC6	CRE	-0.18 [-0.26, -0.11]
Sadness	RC6	IMP	-0.21 [-0.36, -0.06]
Sadness	RC6	EMA	0.55 [0.06, 1.05]
Sadness	RC7	DBA	-1.84 [-2.53, -1.19]
Sadness	RC7	VEN	-0.56 [-0.97, -0.14]
Sadness	RC7	ENT	-0.35 [-0.90, 0.20]
Sadness	RC7	CFE	-0.54 [-0.86, -0.22]
Sadness	RC7	PAX	0.56 [0.16, 0.96]
Sadness	RC7	RAV	-0.36 [-0.69, -0.02]
Sadness	RC7	TES	-0.07 [-0.64, 0.48]
Sadness	RC7	EEK	0.55 [0.15, 0.95]
Sadness	RC7	EDB	1.03 [0.25, 1.85]
Sadness	RC7	SEH	-0.94 [-1.08, -0.80]
Sadness	RC7	DAF	-0.32 [-1.12, 0.49]
Sadness	RC7	EHI	0.07 [-0.67, 0.83]
Sadness	RC7	HAW	-1.01 [-1.88, -0.17]
Sadness	RC7	AH1	1.66 [1.03, 2.33]
Sadness	RC7	AH2	0.18 [-0.04, 0.39]
Sadness	RC7	J01	-0.21 [-0.82, 0.39]
Sadness	RC7	GEM	0.18 [-0.84, 1.16]
Sadness	RC7	SAV	-0.60 [-1.19, 0.01]
Sadness	RC7	CRE	-0.35 [-0.48, -0.23]
Sadness	RC7	IMP	0.23 [0.01, 0.44]
Sadness	RC7	EMA	0.33 [-0.10, 0.77]
Fear	RC1	VEN	0.88 [0.27, 1.51]
Fear	RC1	ENT	0.98 [0.13, 1.84]
Fear	RC1	CFE	1.39 [0.88, 1.89]
Fear	RC1	PAX	4.58 [3.95, 5.21]
Fear	RC1	RAV	0.32 [-0.09, 0.73]
Fear	RC1	TES	5.67 [4.90, 6.45]
Fear	RC1	EDB	3.15 [2.42, 3.90]
Fear	RC1	SEH	0.58 [0.41, 0.75]
Fear	RC1	SES	-0.37 [-0.48, -0.25]
Fear	RC1	DAF	0.82 [-0.42, 2.10]
Fear	RC1	HAW	1.35 [-0.44, 3.19]
Fear	RC1	AH1	0.45 [-0.46, 1.32]
Fear	RC1	AH2	-1.82 [-2.12, -1.54]

Emotion	Factor	Corpus	Mean and CI
Fear	RC1	J01	-0.15 [-1.19, 0.87]
Fear	RC1	SAV	4.84 [3.81, 5.92]
Fear	RC1	CRE	-1.68 [-1.89, -1.46]
Fear	RC2	VEN	1.63 [1.13, 2.13]
Fear	RC2	ENT	0.09 [-0.61, 0.79]
Fear	RC2	CFE	-0.56 [-1.76, 0.62]
Fear	RC2	PAX	3.42 [2.57, 4.28]
Fear	RC2	RAV	3.55 [2.88, 4.25]
Fear	RC2	TES	2.86 [1.76, 4.06]
Fear	RC2	EDB	1.32 [0.56, 2.07]
Fear	RC2	SEH	0.60 [0.41, 0.78]
Fear	RC2	SES	0.06 [-0.06, 0.17]
Fear	RC2	DAF	2.95 [1.09, 4.93]
Fear	RC2	HAW	0.32 [-0.43, 1.09]
Fear	RC2	AH1	-0.31 [-0.66, 0.05]
Fear	RC2	AH2	-1.76 [-2.08, -1.45]
Fear	RC2	J01	0.72 [-0.20, 1.61]
Fear	RC2	SAV	1.17 [0.74, 1.61]
Fear	RC2	CRE	2.59 [2.33, 2.85]
Fear	RC3	VEN	0.39 [-0.33, 1.12]
Fear	RC3	ENT	0.01 [-0.77, 0.81]
Fear	RC3	CFE	-0.60 [-1.37, 0.14]
Fear	RC3	PAX	2.85 [2.10, 3.61]
Fear	RC3	RAV	1.16 [0.56, 1.76]
Fear	RC3	TES	6.44 [5.27, 7.64]
Fear	RC3	EDB	0.03 [-0.90, 0.91]
Fear	RC3	SEH	0.18 [-0.10, 0.46]
Fear	RC3	SES	0.20 [0.16, 0.24]
Fear	RC3	DAF	0.54 [-0.65, 1.78]
Fear	RC3	HAW	0.42 [-1.26, 2.01]
Fear	RC3	AH1	3.34 [2.24, 4.46]
Fear	RC3	AH2	2.05 [1.79, 2.30]
Fear	RC3	J01	0.82 [-0.13, 1.83]
Fear	RC3	SAV	3.31 [2.31, 4.36]
Fear	RC3	CRE	0.94 [0.79, 1.09]
Fear	RC4	VEN	-0.81 [-1.45, -0.19]
Fear	RC4	ENT	0.02 [-0.59, 0.65]
Fear	RC4	CFE	-0.71 [-1.36, -0.08]
Fear	RC4	PAX	2.71 [2.02, 3.42]
Fear	RC4	RAV	-1.01 [-1.79, -0.23]
Fear	RC4	TES	-0.11 [-1.15, 0.91]
Fear	RC4	EDB	-0.62 [-1.37, 0.15]
Fear	RC4	SEH	-0.60 [-0.73, -0.47]
Fear	RC4	SES	-0.57 [-0.70, -0.45]
Fear	RC4	DAF	-2.47 [-3.23, -1.75]
Fear	RC4	HAW	-1.70 [-3.47, -0.07]
Fear	RC4	AH1	0.76 [0.39, 1.13]
Fear	RC4	AH2	0.41 [0.26, 0.56]
Fear	RC4	J01	-1.12 [-2.07, -0.21]
Fear	RC4	SAV	1.03 [0.06, 2.02]
Fear	RC4	CRE	-1.21 [-1.42, -1.01]
Fear	RC5	VEN	0.27 [-0.22, 0.77]
Fear	RC5	ENT	0.15 [-0.37, 0.68]
Fear	RC5	CFE	-0.20 [-0.63, 0.22]
Fear	RC5	PAX	1.67 [1.26, 2.08]
Fear	RC5	RAV	-0.68 [-1.13, -0.25]
Fear	RC5	TES	0.04 [-0.56, 0.63]
Fear	RC5	EDB	-0.75 [-1.36, -0.14]
Fear	RC5	SEH	-0.35 [-0.48, -0.20]
Fear	RC5	SES	-0.22 [-0.33, -0.11]

Emotion	Factor	Corpus	Mean and CI
Fear	RC5	DAF	-0.50 [-1.30, 0.26]
Fear	RC5	HAW	-0.45 [-1.78, 0.83]
Fear	RC5	AH1	-0.18 [-0.71, 0.34]
Fear	RC5	AH2	-3.45 [-3.67, -3.24]
Fear	RC5	J01	0.55 [-0.13, 1.27]
Fear	RC5	SAV	1.93 [1.22, 2.67]
Fear	RC5	CRE	-0.21 [-0.34, -0.08]
Fear	RC6	VEN	0.15 [-0.30, 0.62]
Fear	RC6	ENT	-0.30 [-0.75, 0.14]
Fear	RC6	CFE	-0.09 [-0.43, 0.26]
Fear	RC6	PAX	0.69 [0.33, 1.04]
Fear	RC6	RAV	0.31 [-0.03, 0.65]
Fear	RC6	TES	1.24 [0.69, 1.82]
Fear	RC6	EDB	-0.08 [-0.49, 0.30]
Fear	RC6	SEH	0.08 [-0.02, 0.17]
Fear	RC6	SES	-1.11 [-1.20, -1.02]
Fear	RC6	DAF	0.14 [-0.45, 0.72]
Fear	RC6	HAW	0.05 [-0.72, 0.83]
Fear	RC6	AH1	-1.01 [-1.54, -0.49]
Fear	RC6	AH2	0.38 [0.22, 0.53]
Fear	RC6	J01	0.35 [-0.14, 0.88]
Fear	RC6	SAV	0.54 [0.06, 1.04]
Fear	RC6	CRE	0.08 [0.00, 0.16]
Fear	RC7	VEN	-0.62 [-1.04, -0.20]
Fear	RC7	ENT	-0.10 [-0.66, 0.44]
Fear	RC7	CFE	-1.18 [-1.52, -0.83]
Fear	RC7	PAX	-3.29 [-3.70, -2.91]
Fear	RC7	RAV	-0.68 [-1.02, -0.34]
Fear	RC7	TES	-8.01 [-8.77, -7.29]
Fear	RC7	EDB	-1.08 [-1.54, -0.64]
Fear	RC7	SEH	-0.93 [-1.07, -0.79]
Fear	RC7	SES	-0.34 [-0.43, -0.25]
Fear	RC7	DAF	-1.01 [-1.78, -0.22]
Fear	RC7	HAW	-1.43 [-2.59, -0.30]
Fear	RC7	AH1	-3.75 [-4.32, -3.20]
Fear	RC7	AH2	0.87 [0.65, 1.09]
Fear	RC7	J01	-1.14 [-1.77, -0.53]
Fear	RC7	SAV	-4.33 [-5.13, -3.59]
Fear	RC7	CRE	-1.01 [-1.13, -0.89]
Disgust	RC1	ENT	1.31 [0.47, 2.16]
Disgust	RC1	CFE	1.02 [0.52, 1.52]
Disgust	RC1	PAX	1.57 [1.08, 2.07]
Disgust	RC1	RAV	0.33 [-0.07, 0.74]
Disgust	RC1	TES	-0.36 [-1.01, 0.30]
Disgust	RC1	EDB	1.80 [1.07, 2.57]
Disgust	RC1	SEH	0.78 [0.60, 0.95]
Disgust	RC1	SES	0.72 [0.61, 0.83]
Disgust	RC1	DAF	-0.57 [-1.80, 0.66]
Disgust	RC1	HAW	0.92 [-1.07, 2.93]
Disgust	RC1	AH1	-3.89 [-4.81, -2.98]
Disgust	RC1	AH2	4.87 [4.59, 5.15]
Disgust	RC1	J01	1.96 [0.93, 2.98]
Disgust	RC1	GEM	0.48 [-1.30, 2.26]
Disgust	RC1	SAV	0.25 [-0.59, 1.16]
Disgust	RC1	CRE	-0.37 [-0.57, -0.16]
Disgust	RC1	VDB	-2.11 [-2.36, -1.85]
Disgust	RC2	ENT	0.43 [-0.27, 1.14]
Disgust	RC2	CFE	-0.33 [-1.23, 0.55]
Disgust	RC2	PAX	-0.15 [-0.81, 0.52]
Disgust	RC2	RAV	0.68 [0.00, 1.37]

Emotion	Factor	Corpus	Mean and CI
Disgust	RC2	TES	1.18 [0.15, 2.22]
Disgust	RC2	EDB	0.38 [-0.36, 1.13]
Disgust	RC2	SEH	0.08 [-0.12, 0.27]
Disgust	RC2	SES	0.69 [0.57, 0.81]
Disgust	RC2	DAF	0.12 [-1.23, 1.36]
Disgust	RC2	HAW	0.06 [-0.69, 0.81]
Disgust	RC2	AH1	-1.36 [-1.69, -1.03]
Disgust	RC2	AH2	-1.26 [-1.57, -0.96]
Disgust	RC2	J01	0.83 [0.02, 1.62]
Disgust	RC2	GEM	0.05 [-1.22, 1.40]
Disgust	RC2	SAV	0.93 [0.53, 1.32]
Disgust	RC2	CRE	1.13 [0.86, 1.39]
Disgust	RC2	VDB	-1.01 [-1.28, -0.74]
Disgust	RC3	ENT	-0.50 [-1.29, 0.27]
Disgust	RC3	CFE	-0.89 [-1.59, -0.19]
Disgust	RC3	PAX	0.80 [0.32, 1.28]
Disgust	RC3	RAV	1.46 [0.87, 2.07]
Disgust	RC3	TES	0.68 [-0.27, 1.59]
Disgust	RC3	EDB	-0.27 [-1.21, 0.70]
Disgust	RC3	SEH	-0.79 [-1.11, -0.49]
Disgust	RC3	SES	-0.17 [-0.22, -0.13]
Disgust	RC3	DAF	0.23 [-0.87, 1.35]
Disgust	RC3	HAW	-0.81 [-2.43, 0.72]
Disgust	RC3	AH1	-1.41 [-2.48, -0.37]
Disgust	RC3	AH2	-2.88 [-3.14, -2.61]
Disgust	RC3	J01	0.53 [-0.34, 1.46]
Disgust	RC3	GEM	-0.12 [-1.45, 1.16]
Disgust	RC3	SAV	1.33 [0.50, 2.17]
Disgust	RC3	CRE	0.26 [0.16, 0.36]
Disgust	RC3	VDB	-0.53 [-0.77, -0.30]
Disgust	RC4	ENT	-0.15 [-0.77, 0.49]
Disgust	RC4	CFE	-0.28 [-0.91, 0.37]
Disgust	RC4	PAX	-3.48 [-4.06, -2.90]
Disgust	RC4	RAV	-0.93 [-1.76, -0.11]
Disgust	RC4	TES	-5.44 [-6.41, -4.48]
Disgust	RC4	EDB	-1.79 [-2.60, -0.97]
Disgust	RC4	SEH	-0.75 [-0.89, -0.61]
Disgust	RC4	SES	0.52 [0.39, 0.66]
Disgust	RC4	DAF	-2.15 [-2.84, -1.49]
Disgust	RC4	HAW	0.81 [-0.92, 2.72]
Disgust	RC4	AH1	0.30 [-0.04, 0.64]
Disgust	RC4	AH2	0.17 [0.03, 0.31]
Disgust	RC4	J01	-2.04 [-2.99, -1.11]
Disgust	RC4	GEM	-0.24 [-2.08, 1.59]
Disgust	RC4	SAV	-1.42 [-2.33, -0.50]
Disgust	RC4	CRE	-1.59 [-1.79, -1.38]
Disgust	RC4	VDB	0.02 [-0.19, 0.24]
Disgust	RC5	ENT	-0.18 [-0.70, 0.35]
Disgust	RC5	CFE	0.79 [0.38, 1.20]
Disgust	RC5	PAX	1.78 [1.47, 2.09]
Disgust	RC5	RAV	0.22 [-0.23, 0.67]
Disgust	RC5	TES	0.40 [-0.06, 0.86]
Disgust	RC5	EDB	0.73 [0.13, 1.34]
Disgust	RC5	SEH	-0.92 [-1.07, -0.77]
Disgust	RC5	SES	0.22 [0.12, 0.33]
Disgust	RC5	DAF	-0.37 [-1.11, 0.33]
Disgust	RC5	HAW	-0.40 [-1.54, 0.68]
Disgust	RC5	AH1	-1.00 [-1.51, -0.50]
Disgust	RC5	AH2	0.67 [0.48, 0.85]
Disgust	RC5	J01	0.96 [0.35, 1.57]

Emotion	Factor	Corpus	Mean and CI
Disgust	RC5	GEM	0.67 [-0.29, 1.66]
Disgust	RC5	SAV	1.23 [0.69, 1.80]
Disgust	RC5	CRE	-0.05 [-0.18, 0.08]
Disgust	RC5	VDB	0.90 [0.70, 1.10]
Disgust	RC6	ENT	0.41 [-0.03, 0.88]
Disgust	RC6	CFE	1.27 [0.95, 1.60]
Disgust	RC6	PAX	1.47 [1.19, 1.75]
Disgust	RC6	RAV	1.51 [1.18, 1.84]
Disgust	RC6	TES	4.90 [4.44, 5.39]
Disgust	RC6	EDB	0.32 [-0.05, 0.69]
Disgust	RC6	SEH	0.41 [0.31, 0.50]
Disgust	RC6	SES	-0.61 [-0.70, -0.51]
Disgust	RC6	DAF	0.84 [0.34, 1.33]
Disgust	RC6	HAW	0.79 [-0.18, 1.76]
Disgust	RC6	AH1	-1.48 [-2.00, -0.98]
Disgust	RC6	AH2	1.52 [1.37, 1.66]
Disgust	RC6	J01	0.88 [0.36, 1.39]
Disgust	RC6	GEM	0.76 [-0.32, 1.85]
Disgust	RC6	SAV	1.43 [1.00, 1.87]
Disgust	RC6	CRE	0.45 [0.37, 0.52]
Disgust	RC6	VDB	0.36 [0.25, 0.49]
Disgust	RC7	ENT	-0.19 [-0.73, 0.36]
Disgust	RC7	CFE	-0.81 [-1.13, -0.50]
Disgust	RC7	PAX	-0.59 [-0.90, -0.28]
Disgust	RC7	RAV	-0.25 [-0.58, 0.09]
Disgust	RC7	TES	-0.62 [-1.21, -0.02]
Disgust	RC7	EDB	-1.18 [-1.68, -0.72]
Disgust	RC7	SEH	-1.18 [-1.32, -1.03]
Disgust	RC7	SES	-0.42 [-0.51, -0.33]
Disgust	RC7	DAF	-1.63 [-2.35, -0.93]
Disgust	RC7	HAW	-0.47 [-1.42, 0.52]
Disgust	RC7	AH1	0.79 [0.23, 1.36]
Disgust	RC7	AH2	-0.32 [-0.52, -0.12]
Disgust	RC7	J01	0.31 [-0.29, 0.94]
Disgust	RC7	GEM	0.14 [-0.86, 1.19]
Disgust	RC7	SAV	-1.63 [-2.22, -1.04]
Disgust	RC7	CRE	-0.37 [-0.48, -0.26]
Disgust	RC7	VDB	-1.51 [-1.68, -1.33]
Surprise	RC1	ENT	-0.09 [-0.94, 0.77]
Surprise	RC1	CFE	-0.34 [-0.88, 0.19]
Surprise	RC1	PAX	6.53 [5.71, 7.38]
Surprise	RC1	RAV	-0.15 [-0.57, 0.27]
Surprise	RC1	TES	2.72 [2.01, 3.44]
Surprise	RC1	SEH	0.84 [0.64, 1.04]
Surprise	RC1	SES	0.16 [0.05, 0.27]
Surprise	RC1	DAF	0.41 [-0.71, 1.49]
Surprise	RC1	HAW	0.09 [-1.53, 1.65]
Surprise	RC1	AH1	0.54 [-0.28, 1.35]
Surprise	RC1	AH2	-1.36 [-1.70, -1.01]
Surprise	RC1	SAV	3.77 [2.64, 4.91]
Surprise	RC2	ENT	0.77 [0.07, 1.47]
Surprise	RC2	CFE	1.15 [0.17, 2.15]
Surprise	RC2	PAX	5.10 [4.08, 6.20]
Surprise	RC2	RAV	1.16 [0.50, 1.87]
Surprise	RC2	TES	4.50 [3.36, 5.67]
Surprise	RC2	SEH	1.59 [1.39, 1.79]
Surprise	RC2	SES	0.40 [0.28, 0.51]
Surprise	RC2	DAF	1.40 [-0.12, 2.91]
Surprise	RC2	HAW	0.39 [-0.44, 1.24]
Surprise	RC2	AH1	0.12 [-0.21, 0.44]

Emotion	Factor	Corpus	Mean and CI
Surprise	RC2	AH2	-1.27 [-1.68, -0.85]
Surprise	RC2	SAV	1.56 [1.11, 2.01]
Surprise	RC3	ENT	0.33 [-0.47, 1.11]
Surprise	RC3	CFE	0.81 [0.08, 1.55]
Surprise	RC3	PAX	5.57 [4.52, 6.68]
Surprise	RC3	RAV	1.63 [0.94, 2.31]
Surprise	RC3	TES	4.02 [2.92, 5.14]
Surprise	RC3	SEH	2.21 [1.87, 2.56]
Surprise	RC3	SES	-0.06 [-0.10, -0.01]
Surprise	RC3	DAF	1.16 [-0.08, 2.41]
Surprise	RC3	HAW	1.47 [-0.46, 3.35]
Surprise	RC3	AH1	3.28 [2.23, 4.33]
Surprise	RC3	AH2	5.07 [4.67, 5.47]
Surprise	RC3	SAV	5.07 [3.92, 6.27]
Surprise	RC4	ENT	-0.08 [-0.70, 0.54]
Surprise	RC4	CFE	-0.33 [-1.02, 0.33]
Surprise	RC4	PAX	1.87 [1.03, 2.76]
Surprise	RC4	RAV	-1.52 [-2.34, -0.72]
Surprise	RC4	TES	1.32 [0.27, 2.34]
Surprise	RC4	SEH	-0.04 [-0.18, 0.11]
Surprise	RC4	SES	0.52 [0.39, 0.65]
Surprise	RC4	DAF	-2.22 [-2.99, -1.49]
Surprise	RC4	HAW	-0.25 [-1.89, 1.35]
Surprise	RC4	AH1	-0.03 [-0.36, 0.29]
Surprise	RC4	AH2	1.79 [1.59, 2.00]
Surprise	RC4	SAV	1.24 [0.24, 2.28]
Surprise	RC5	ENT	-0.24 [-0.78, 0.30]
Surprise	RC5	CFE	-0.96 [-1.44, -0.49]
Surprise	RC5	PAX	3.36 [2.82, 3.92]
Surprise	RC5	RAV	-1.21 [-1.69, -0.73]
Surprise	RC5	TES	2.43 [1.90, 2.98]
Surprise	RC5	SEH	0.37 [0.22, 0.53]
Surprise	RC5	SES	-0.23 [-0.34, -0.13]
Surprise	RC5	DAF	-1.09 [-1.88, -0.32]
Surprise	RC5	HAW	-1.08 [-2.45, 0.21]
Surprise	RC5	AH1	0.65 [0.13, 1.17]
Surprise	RC5	AH2	-2.03 [-2.30, -1.77]
Surprise	RC5	SAV	1.49 [0.73, 2.26]
Surprise	RC6	ENT	0.32 [-0.14, 0.77]
Surprise	RC6	CFE	1.60 [1.26, 1.93]
Surprise	RC6	PAX	3.37 [2.91, 3.85]
Surprise	RC6	RAV	2.12 [1.79, 2.47]
Surprise	RC6	TES	6.24 [5.71, 6.77]
Surprise	RC6	SEH	1.19 [1.09, 1.30]
Surprise	RC6	SES	0.03 [-0.06, 0.11]
Surprise	RC6	DAF	1.46 [0.93, 1.97]
Surprise	RC6	HAW	1.74 [0.78, 2.72]
Surprise	RC6	AH1	1.88 [1.43, 2.33]
Surprise	RC6	AH2	2.43 [2.24, 2.62]
Surprise	RC6	SAV	1.79 [1.28, 2.30]
Surprise	RC7	ENT	-0.29 [-0.84, 0.25]
Surprise	RC7	CFE	-2.29 [-2.68, -1.92]
Surprise	RC7	PAX	-5.75 [-6.31, -5.21]
Surprise	RC7	RAV	-1.30 [-1.66, -0.95]
Surprise	RC7	TES	-5.48 [-6.20, -4.79]
Surprise	RC7	SEH	-2.52 [-2.68, -2.36]
Surprise	RC7	SES	-1.32 [-1.41, -1.23]
Surprise	RC7	DAF	-2.32 [-3.14, -1.57]
Surprise	RC7	HAW	-2.08 [-3.28, -0.91]
Surprise	RC7	AH1	-2.25 [-2.75, -1.78]

Emotion	Factor	Corpus	Mean and CI
Surprise	RC7	AH2	-4.35 [-4.63, -4.07]
Surprise	RC7	SAV	-4.53 [-5.33, -3.76]

Supplementary Table 9 | Mean estimates with credible interval displayed in Figure 2c. A subset (Anger, RC2) is plotted in Figure 2d.

	Model name	WAIC	SE
1	big	93162.83	447.25
2	corpus	126569.85	412.09
3	in-group	135354.32	414.87
4	interaction	135354.32	414.87
5	country	138407.08	407.81
6	language	139666.21	402.96
7	base	154974.72	354.21
8	null	182532.85	164.40

Supplementary Table 10 | WAIC values for each model reported in Figure 3a–b.

	NEU	ANG	DIS	FER	HAP	SAD	SUR
NEU	46.05	8.19	14.21	12.14	8.94	8.33	2.14
ANG	6.56	51.75	10.89	6.93	18.58	1.80	3.49
DIS	15.33	14.41	25.98	12.95	17.22	7.03	7.08
FER	16.58	8.02	17.20	26.90	14.68	10.17	6.45
HAP	10.05	23.82	13.80	9.52	28.76	4.51	9.54
SAD	18.36	2.26	12.51	15.86	5.18	41.04	4.79
SUR	5.40	10.52	10.31	9.54	15.54	4.20	44.50

Supplementary Table 11 | Confusion matrix (UAR) for the base model reported in Figure 3e.

	NEU	ANG	DIS	FER	HAP	SAD	SUR
NEU	54.27	5.76	12.49	8.25	9.17	7.90	2.16
ANG	5.86	60.01	9.21	5.44	13.90	1.67	3.90
DIS	14.06	11.26	37.43	10.81	11.51	7.35	7.57
FER	10.25	8.17	12.44	39.14	14.13	9.58	6.28
HAP	11.74	18.78	9.87	6.50	41.65	4.53	6.93
SAD	13.49	3.05	11.55	15.17	5.15	48.28	3.31
SUR	5.11	8.60	7.68	8.90	11.17	4.20	54.34

Supplementary Table 12 | Confusion matrix (UAR) for the in-group model reported in Figure 3e.

	NEU	ANG	DIS	FER	HAP	SAD	SUR
NEU	73.77	3.33	6.44	3.63	5.35	6.37	1.10
ANG	3.42	79.05	5.16	2.83	5.57	0.96	3.01
DIS	8.01	7.15	64.27	5.37	6.08	5.82	3.29
FER	5.49	4.50	5.91	62.19	6.91	9.81	5.19
HAP	8.23	9.08	7.00	7.54	60.53	2.02	5.60
SAD	8.40	1.55	4.89	6.10	2.23	75.95	0.88
SUR	1.87	5.49	4.53	6.27	6.17	0.99	74.68

Supplementary Table 13 | Confusion matrix (UAR) for the big model reported in Figure 3e.

Predicted emotion	Level	Mean	SD
ANG	corpus	10.87	1.72
ANG	culture	21.41	2.34
ANG	global	16.32	3.69
ANG	sex	19.77	4.38
ANG	speaker	31.62	1.59
DIS	corpus	14.73	1.91
DIS	culture	19.78	3.82
DIS	global	17.99	3.42
DIS	sex	12.13	2.83
DIS	speaker	35.37	2.53
FER	corpus	10.58	2.33
FER	culture	21.13	2.44
FER	global	21.20	3.73
FER	sex	13.87	3.28
FER	speaker	33.22	2.07
HAP	corpus	12.05	1.33
HAP	culture	17.47	2.05
HAP	global	24.52	4.77
HAP	sex	18.50	5.08
HAP	speaker	27.46	1.58
SAD	corpus	10.66	1.69
SAD	culture	24.86	2.79
SAD	global	17.90	3.76
SAD	sex	12.74	4.51
SAD	speaker	33.84	2.00
SUR	corpus	8.16	3.30
SUR	culture	28.65	4.03
SUR	global	16.58	3.95
SUR	sex	19.26	4.68
SUR	speaker	27.35	2.31

Supplementary Table 14 | Mean and standard deviation of the contribution of each level for the model prediction as depicted in Figure 4a.

Level	Factor	Mean of SD	SD of SD
speaker	RC1	2.28	0.35
culture	RC1	2.22	0.71
speaker	RC3	2.07	0.36
speaker	RC2	2.00	0.56
culture	RC3	1.78	0.72
culture	RC2	1.70	0.75
speaker	RC7	1.56	0.25
speaker	RC4	1.28	0.27
culture	RC4	1.22	0.50
culture	RC5	1.20	0.22
culture	RC7	1.13	0.26
speaker	RC5	1.01	0.23
sex	RC1	1.00	0.36
sex	RC3	0.90	0.28
global	RC1	0.82	0.16
sex	RC2	0.81	0.32
culture	RC6	0.80	0.21
global	RC2	0.74	0.22
global	RC3	0.74	0.10
sex	RC7	0.72	0.25
speaker	RC6	0.70	0.09
global	RC7	0.67	0.18
global	RC4	0.55	0.11
global	RC5	0.50	0.05
global	RC6	0.49	0.10
sex	RC4	0.48	0.14
sex	RC6	0.48	0.14
sex	RC5	0.44	0.06

Supplementary Table 15 | The average standard deviation of all coefficients by the acoustic factor as depicted in Figure 4b (left).

Level	Factor	Mean of SD	SD of SD
culture	SAD	1.86	0.79
speaker	SUR	1.81	0.64
speaker	SAD	1.71	0.80
speaker	ANG	1.63	0.76
culture	ANG	1.62	0.88
culture	SUR	1.61	0.72
speaker	FER	1.55	0.59
speaker	DIS	1.40	0.50
culture	FER	1.38	0.53
speaker	HAP	1.24	0.49
culture	HAP	1.19	0.40
culture	DIS	0.96	0.30
sex	ANG	0.82	0.41
sex	SUR	0.77	0.30
sex	HAP	0.74	0.29
global	SUR	0.72	0.17
global	ANG	0.71	0.23
global	HAP	0.70	0.21
sex	DIS	0.69	0.39
global	SAD	0.61	0.17
sex	SAD	0.58	0.24
global	FER	0.57	0.12
global	DIS	0.55	0.13
sex	FER	0.53	0.14

Supplementary Table 16 | The average standard deviation of all coefficients by the emotion as depicted in Figure 4b (right).

	ANG	DIS	FER	HAP	SAD	SUR
ANG	1.00	0.48	0.44	0.52	0.01	0.47
DIS	0.48	1.00	0.43	0.45	0.25	0.44
FER	0.44	0.43	1.00	0.65	0.19	0.67
HAP	0.52	0.45	0.65	1.00	0.04	0.62
SAD	0.01	0.25	0.19	0.04	1.00	0.19
SUR	0.47	0.44	0.67	0.62	0.19	1.00

Supplementary Table 17 | Correlation matrix for mappings of all emotions correlated with each other as depicted in Figure 4c (top, left).

	ANG	DIS	FER	HAP	SAD	SUR
ANG	0.98	0.58	0.63	0.78	0.33	0.55
DIS	0.58	0.84	0.51	0.62	0.22	0.51
FER	0.63	0.51	0.99	0.82	0.17	0.83
HAP	0.78	0.62	0.82	0.99	0.05	0.80
SAD	0.33	0.22	0.17	0.05	0.99	0.23
SUR	0.55	0.51	0.83	0.80	0.23	0.98

Supplementary Table 18 | Correlation matrix of correlations between the global mapping with sex as depicted in Figure 4c (top, right).

	ANG	DIS	FER	HAP	SAD	SUR
ANG	0.42	0.26	0.28	0.30	0.20	0.27
DIS	0.26	0.52	0.29	0.28	0.24	0.28
FER	0.28	0.29	0.58	0.45	0.26	0.46
HAP	0.30	0.28	0.45	0.67	0.23	0.44
SAD	0.20	0.24	0.26	0.23	0.43	0.27
SUR	0.27	0.28	0.46	0.44	0.27	0.69

Supplementary Table 19 | Correlation matrix of correlations between the global mapping with culture as depicted in Figure 4c (bottom, left).

	ANG	DIS	FER	HAP	SAD	SUR
ANG	0.32	0.18	0.21	0.23	0.14	0.19
DIS	0.18	0.33	0.22	0.23	0.15	0.20
FER	0.21	0.22	0.45	0.32	0.18	0.30
HAP	0.23	0.23	0.32	0.56	0.19	0.30
SAD	0.14	0.15	0.18	0.19	0.33	0.16
SUR	0.19	0.20	0.30	0.30	0.16	0.41

Supplementary Table 20 | Correlation matrix of correlations between the global mapping with speaker as depicted in Figure 4c (bottom, right).

Supplementary Discussion

Acted vs. non-acted speech corpora

Corpora of emotional prosody are often divided into "acted" and "spontaneous" corpora. However, the boundary between both groups is often not so clear: Spontaneous emotional corpora are rarely really "spontaneous". For example, the improvisation fragments in the popular IEMOCAP corpus[47] are often referred to as spontaneous recordings[48]; however, the recorded responses are within an acting game and might differ from expression in daily life. Generally, in both groups, the speakers are aware they are being recorded, which might affect their response. Furthermore, not all "acted" corpora rely on actors and many "spontaneous" corpora use professional actors[47,49,50].

Nonetheless, both groups differ in two key aspects. First, "acted" corpora — in contrast to "spontaneous" corpora — have "ground-truth" labels, since it is known which emotion should be depicted. Such labels are missing in spontaneous corpora. Instead, to get an estimate of the expressed emotion in spontaneous corpora, each fragment needs to be annotated manually. Here, a label with high agreement could serve as a label for classification, but the agreement across annotators is often low[51]. In Supplementary Discussion 3, we outline why validation is not so straightforward.

The second key difference between both groups is that speakers in acted corpora are given only one emotion label per utterance, whereas spontaneous utterances tend to contain blends of emotions[51,52]. While this in itself is certainly a better approximation of human emotion communication — often a mix rather than just one emotion is expressed — it is problematic for two reasons. First, to the best of our knowledge, there is no corpus available that annotated enough responses from different participants for the same stimulus such that one can analyze blends of emotion. The second issue is that emotion recognition is often stated as a classification problem in which only one emotion needs to be selected, ignoring the possibility that the recordings might contain blends of emotions.

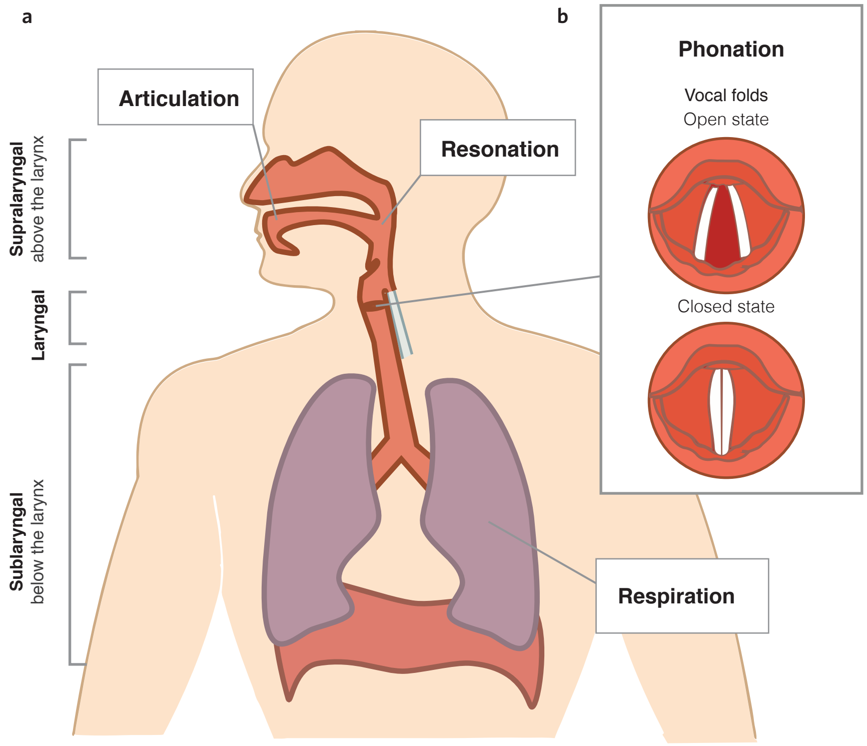

Supplementary Figure 7 | Vocal apparatus. **a** Four subsystems involved in the production of speech: respiration (in- and exhalation), phonation (production of fundamental frequency, see **b**), resonation (creation of formants), and articulation (shifting formants). **b** The vocal folds in the open and closed state. Illustrations are adapted from Gordon Betts *et al.* [53].

The production of speech prosody

There are multiple definitions of speech prosody. We define speech prosody as variations in pitch, loudness, timing, and voice quality. Speech prosody is the product of human speech production that involves four subsystems. All subsystems are illustrated in Fig. 7a. The respiratory system is responsible for the in- and exhalation of air, which is needed to produce pressure. This subsystem is also responsible for loudness-related features. During phonation, the vocal folds rapidly open and close (Fig. 7b for a depiction of the vocal folds in an open and closed state). This subsystem is responsible for pitch production: the number of vibrations of the vocal folds is called the fundamental frequency. During resonation, formants are created and can be shifted during articulation. So the last two subsystems are mainly responsible for the spectral content of the sound.

Validation of corpora of emotional prosody

Estimating the validity of emotional speech usually involves external raters who identify or rate the intended emotion. However, the validation of corpora is not straightforward, as there is no consensus on cutoff values to call a corpus "valid" — such as the minimal number of ratings per stimulus. Furthermore, the concept of validation builds upon the controversial assumption that emotions can be recognized at above chance level from the voice. Thus, corpus validation might not lead to the selection of valid depictions of an emotion, but instead, it might lead to the selection of prototypical depictions. Heedful of these considerations, we will not evaluate the validity of the emotional expression across datasets.

References

1. Laukka, P. & Elfenbein, H. A. Cross-Cultural Emotion Recognition and In-Group Advantage in Vocal Expression: A Meta-Analysis. en. *Emotion Review,* 175407391989729. ISSN: 1754-0739, 1754-0747. doi:10/ggk846 (2020).
2. Juslin, P. N., Laukka, P. & Bänziger, T. The Mirror to Our Soul? Comparisons of Spontaneous and Posed Vocal Expression of Emotion. *Journal of Nonverbal Behavior* **42,** 1–40. doi:10/gcjm2b (2017).
3. Anagnostopoulos, C.-N., Iliou, T. & Giannoukos, I. Features and Classifiers for Emotion Recognition from Speech: A Survey from 2000 to 2011. en. *Artificial Intelligence Review* **43,** 155–177. ISSN: 0269-2821, 1573-7462. doi:10/ggcgrm (2015).
4. El Ayadi, M., Kamel, M. S. & Karray, F. Survey on Speech Emotion Recognition: Features, Classification Schemes, and Databases. en. *Pattern Recognition* **44,** 572–587. ISSN: 00313203. doi:10/fjdzpq (2011).
5. Ververidis, D. & Kotropoulos, C. Emotional Speech Recognition: Resources, Features, and Methods. *Speech Communication* **48,** 1162–1181. doi:10/dtmxsh (2006).
6. Pittermann, J., Pittermann, A. & Minker, W. *Handling Emotions in Human-Computer Dialogues* doi:10.1007/978-90-481-3129-7 (Springer Netherlands, 2010).
7. Lassalle, A. *et al.* The EU-Emotion Voice Database. *Behavior Research Methods* **51,** 493–506. doi:10.3758/s13428-018-1048-1 (Apr. 2018).
8. Swain, M., Routray, A. & Kabisatpathy, P. Databases, features and classifiers for speech emotion recognition: a review. *International Journal of Speech Technology* **21,** 93–120. doi:10.1007/s10772-018-9491-z (Jan. 2018).
9. Navas, E., Hernáez, I., Castelruiz, A. & Luengo, I. *Obtaining and Evaluating an Emotional Database for Prosody Modelling in Standard Basque* en. in *Text, Speech and Dialogue* (eds Sojka, P., Kopeček, I. & Pala, K.) (Springer, Berlin, Heidelberg, 2004), 393–400. ISBN: 978-3-540-30120-2. doi:10/bzdvmt.
10. Saratxaga, I., Navas, E., Hernáez, I. & Luengo, I. *Designing and Recording an Emotional Speech Database for Corpus Based Synthesis in Basque* en. in *Proceedings of the Fifth International Conference on Language Resources and Evaluation (LREC'06)* (European Language Resources Association (ELRA), 2006), 4.
11. Gournay, P., Lahaie, O. & Lefebvre, R. *A Canadian French Emotional Speech Dataset* en. in *Proceedings of the 9th ACM Multimedia Systems Conference* (ACM, Amsterdam Netherlands, 2018), 399–402. ISBN: 978-1-4503-5192-8. doi:10/gj4hht.
12. Cao, H. *et al.* CREMA-D: Crowd-Sourced Emotional Multimodal Actors Dataset. *IEEE Transactions on Affective Computing* **5,** 377–390. ISSN: 1949-3045. doi:10/ggjdbh (2014).
13. Battocchi, A., Pianesi, F. & Goren-Bar, D. in *Intelligent Technologies for Interactive Entertainment* (eds Hutchison, D. *et al.*) 303–306 (Springer Berlin Heidelberg, Berlin, Heidelberg, 2005). doi:10.1007/11590323_39.
14. Hadjadji, I., Falek, L., Demri, L. & Teffahi, H. *Emotion Recognition in Arabic Speech* in *2019 International Conference on Advanced Electrical Engineering (ICAEE)* (2019), 1–5. doi:10.1109/ICAEE47123.2019.9014809.
15. Burkhardt, F., Paeschke, A., Rolfes, M., Sendlmeier, W. & Weiss, B. *A Database of German Emotional Speech* en. in *INTERSPEECH* (Lisbon, Portugal, 2005), 4.
16. Altrov, R. & Pajupuu, H. *Estonian Emotional Speech Corpus: Theoretical Base and Implementation* in *Conference: 4th International Workshop on Corpora for Research on EMOTION SENTIMENT & SOCIAL SIGNALS ES*^{3} (Istanbul, Turkey, 2012).
17. Nagels, L. *et al. Vocal Emotion Recognition in School-Age Children: Normative Data for the EmoHI Test* en. Preprint (PeerJ Preprints, 2019). doi:10.7287/peerj.preprints.27921v1.
18. Lee, S., Yildirim, S., Kazemzadeh, A. & Narayanan, S. *An Articulatory Study of Emotional Speech Production* en. in *INTERSPEECH* (Lisbon, Portugal, 2005), 4.
19. Martin, O., Kotsia, I., Macq, B. & Pitas, I. *The eNTERFACE’05 Audio-Visual Emotion Database* in *22nd International Conference on Data Engineering Workshops (ICDEW'06)* (IEEE, Atlanta, GA, USA, 2006), 8–8. ISBN: 978-0-7695-2571-6. doi:10/cckzj4.
20. Ykhlef, F. *et al. Towards Building an Emotional Speech Corpus of Algerian Dialect: Criteria and Preliminary Assessment Results* in *2019 International Conference on Advanced Electrical Engineering (ICAEE)* (2019), 1–6. doi:10/ghjxg4.
21. Bänziger, T., Mortillaro, M. & Scherer, K. R. Introducing the Geneva Multimodal Expression Corpus for Experimental Research on Emotion Perception. en. *Emotion* **12,** 1161–1179. ISSN: 1931-1516, 1528-3542. doi:10/bkgz8f (2012).

22. Hawk, S. T., van Kleef, G. A., Fischer, A. H. & van der Schalk, J. "Worth a Thousand Words": Absolute and Relative Decoding of Nonlinguistic Affect Vocalizations. en. *Emotion* **9,** 293–305. ISSN: 1931-1516, 1528-3542. doi:10/d8k9ps (2009).
23. Busso, C. *et al.* MSP-IMPROV: An Acted Corpus of Dyadic Interactions to Study Emotion Perception. *IEEE Transactions on Affective Computing* **8,** 67–80. ISSN: 1949-3045. doi:10/f9t4sr (2017).
24. Juslin, P. N. & Laukka, P. Impact of Intended Emotion Intensity on Cue Utilization and Decoding Accuracy in Vocal Expression of Emotion. en. *Emotion* **1,** 381–412. ISSN: 1931-1516, 1528-3542. doi:10/dzptgs (2001).
25. Pell, M. D., Paulmann, S., Dara, C., Alasseri, A. & Kotz, S. A. Factors in the Recognition of Vocally Expressed Emotions: A Comparison of Four Languages. *Journal of Phonetics* **37,** 417–435. doi:10/bwzhnd (2009).
26. Livingstone, S. R. & Russo, F. A. The Ryerson Audio-Visual Database of Emotional Speech and Song (RAVDESS): A Dynamic, Multimodal Set of Facial and Vocal Expressions in North American English. en. *PLOS ONE* **13** (ed Najbauer, J.) e0196391. ISSN: 1932-6203. doi:10/gd8gt8 (2018).
27. Haq, S. & Jackson, P. in (ed Wang, W.) 398–423 (IGI Global, Hershey PA, 2010).
28. Koolagudi, S. G., Maity, S., Kumar, V. A., Chakrabarti, S. & Rao, K. S. *IITKGP-SESC: Speech Database for Emotion Analysis* en. in *Contemporary Computing* (eds Ranka, S. *et al.*) (Springer, Berlin, Heidelberg, 2009), 485–492. ISBN: 978-3-642-03547-0. doi:10/ckb3nt.
29. Koolagudi, S. G., Reddy, R., Yadav, J. & Rao, K. S. *IITKGP-SEHSC : Hindi Speech Corpus for Emotion Analysis* in *2011 International Conference on Devices and Communications (ICDeCom)* (2011), 1–5. doi:10/c9xq5m.
30. Pichora-Fuller, M. K. & Dupuis, K. *Toronto Emotional Speech Set (TESS)* 2020. doi:10.5683/SP2/E8H2MF.
31. Adigwe, A., Tits, N., Haddad, K. E., Ostadabbas, S. & Dutoit, T. The Emotional Voices Database: Towards Controlling the Emotion Dimension in Voice Generation Systems. *arXiv:1806.09514 [cs, eess]*. arXiv: 1806.09514 [cs, eess] (2018).
32. Laukka, P. *et al.* The Expression and Recognition of Emotions in the Voice across Five Nations: A Lens Model Analysis Based on Acoustic Features. *Journal of Personality and Social Psychology* **111,** 686–705. doi:10/f3tfdg (2016).
33. Andics, A. & Faragó, T. en. in *The Oxford Handbook of Voice Perception* 362–392 (Oxford University Press, 2018). ISBN: 978-0-19-874318-7. doi:10.1093/oxfordhb/9780198743187.013.16.
34. Rabinov, C. R., Kreiman, J., Gerratt, B. R. & Bielamowicz, S. Comparing Reliability of Perceptual Ratings of Roughness and Acoustic Measures of Jitter. en. *Journal of Speech, Language, and Hearing Research* **38,** 26–32. ISSN: 1092-4388, 1558-9102. doi:10/ghtztc (Feb. 1995).
35. Coleman, R. O. Male and Female Voice Quality and Its Relationship to Vowel Formant Frequencies. *Journal of Speech and Hearing Research* **14,** 565–577. doi:10.1044/jshr.1403.565 (Sept. 1971).
36. Deal, R. E. & Emanuel, F. W. Some Waveform and Spectral Features of Vowel Roughness. *Journal of Speech and Hearing Research* **21,** 250–264. doi:10.1044/jshr.2102.250 (June 1978).
37. Wayland, R., Gargash, S. & Longman, A. Acoustic and Perceptual Investigation of Breathy Voice. *The Journal of the Acoustical Society of America* **97,** 3364–3364. ISSN: 0001-4966. doi:10/fwpgb3 (May 1995).
38. Leino, T. Long-Term Average Spectrum in Screening of Voice Quality in Speech: Untrained Male University Students. en. *Journal of Voice* **23,** 671–676. ISSN: 08921997. doi:10/cvnt93 (Nov. 2009).
39. Sluijter, A. M. C. & van Heuven, V. J. Spectral Balance as an Acoustic Correlate of Linguistic Stress. en. *The Journal of the Acoustical Society of America* **100,** 2471–2485. ISSN: 0001-4966. doi:10/c8rrnq (Oct. 1996).
40. Tamarit, L., Goudbeek, M. & Scherer, K. *Spectral Slope Measurements in Emotionally Expressive Speech* en. in *Speech Analysis and Processing for Knowledge Discovery* (2008), 4. ISBN: 978-87-92328-00-7.
41. Burger, B., Thompson, M. R., Luck, G., Saarikallio, S. & Toiviainen, P. Influences of Rhythm- and Timbre-Related Musical Features on Characteristics of Music-Induced Movement. English. *Frontiers in Psychology* **4.** ISSN: 1664-1078. doi:10/gbfpv2 (2013).
42. Alías, F., Socoró, J. & Sevillano, X. A Review of Physical and Perceptual Feature Extraction Techniques for Speech, Music and Environmental Sounds. en. *Applied Sciences* **6,** 143. ISSN: 2076-3417. doi:10/ghvnff (May 2016).
43. Nordström, H., Laukka, P., Pell, M., Stockholms universitet & Samhällsvetenskapliga fakulteten. *Emotional Communication in the Human Voice* English. ISBN: 978-91-7797-736-0 (2019).
44. Watanabe, S. Asymptotic Equivalence of Bayes Cross Validation and Widely Applicable Information Criterion in Singular Learning Theory. *Journal of Machine Learning Research* **11,** 3571–3594 (2010).
45. McElreath, R. *Statistical Rethinking: A Bayesian Course with Examples in R and STAN* Second. ISBN: 978-0-367-13991-9 (Chapman and Hall, 2020).
46. Team, S. D. *Stan Modeling Language Users Guide and Reference Manual* version 2.26. 2019.
47. Busso, C. *et al.* IEMOCAP: Interactive Emotional Dyadic Motion Capture Database. en. *Language Resources and Evaluation* **42,** 335–359. ISSN: 1574-020X, 1574-0218. doi:10/bcbjzg (2008).
48. Chenchah, F. & Lachiri, Z. Speech Emotion Recognition in Acted and Spontaneous Context. en. *Procedia Computer Science. The 6th International Conference on Intelligent Human Computer Interaction, IHCI 2014* **39,** 139–145. ISSN: 1877-0509. doi:10/ghrk23 (2014).

49. Perepelkina, O., Kazimirova, E. & Konstantinova, M. in *Speech and Computer* 501–510 (Springer International Publishing, 2018). doi:10.1007/978-3-319-99579-3_52.

50. Metallinou, A. *et al.* The USC CreativeIT database of multimodal dyadic interactions: from speech and full body motion capture to continuous emotional annotations. *Language Resources and Evaluation* **50,** 497–521. doi:10.1007/s10579-015-9300-0 (Apr. 2015).

51. Vogt, T., André, E. & Wagner, J. en. in *Affect and Emotion in Human-Computer Interaction: From Theory to Applications* (eds Peter, C. & Beale, R.) 75–91 (Springer, Berlin, Heidelberg, 2008). ISBN: 978-3-540-85099-1. doi:10.1007/978-3-540-85099-1_7.

52. Cowie, R. & Cornelius, R. R. Describing the Emotional States That Are Expressed in Speech. en. *Speech Communication* **40,** 5–32. ISSN: 01676393. doi:10/cd5x56 (2003).

53. Gordon Betts, J. *et al.* *Anatomy and physiology* en (June 2013).
