# Supplementary material for: Modelling individual and cross-cultural variation in the mapping of emotions to speech prosody
Source: Nat Hum Behav. 2023 Jan 16;7(3):386–96. doi: 10.1038/s41562-022-01505-5 (PMC10038802; doi:10.1038/s41562-022-01505-5)
Supplement: Supplementary file 2 — Reporting Summary [file 41562_2022_1505_MOESM2_ESM.pdf]

Corresponding author(s): Pol van Rijn

Last updated by author(s): Nov 24, 2022

Reporting Summary

Nature Portfolio wishes to improve the reproducibility of the work that we publish. This form provides structure for consistency and transparency in reporting. For further information on Nature Portfolio policies, see our Editorial Policies and the Editorial Policy Checklist.

Statistics

For all statistical analyses, confirm that the following items are present in the figure legend, table legend, main text, or Methods section.

n/a	Confirmed	
☐	☒	The exact sample size (n) for each experimental group/condition, given as a discrete number and unit of measurement
☐	☒	A statement on whether measurements were taken from distinct samples or whether the same sample was measured repeatedly
☐	☒	The statistical test(s) used AND whether they are one- or two-sided<br>*Only common tests should be described solely by name; describe more complex techniques in the Methods section.*
☐	☒	A description of all covariates tested
☐	☒	A description of any assumptions or corrections, such as tests of normality and adjustment for multiple comparisons
☐	☒	A full description of the statistical parameters including central tendency (e.g. means) or other basic estimates (e.g. regression coefficient) AND variation (e.g. standard deviation) or associated estimates of uncertainty (e.g. confidence intervals)
☐	☒	For null hypothesis testing, the test statistic (e.g. F, t, r) with confidence intervals, effect sizes, degrees of freedom and P value noted<br>*Give P values as exact values whenever suitable.*
☐	☒	For Bayesian analysis, information on the choice of priors and Markov chain Monte Carlo settings
☐	☒	For hierarchical and complex designs, identification of the appropriate level for tests and full reporting of outcomes
☒	☐	Estimates of effect sizes (e.g. Cohen's d, Pearson's r), indicating how they were calculated

Our web collection on statistics for biologists contains articles on many of the points above.

Software and code

Policy information about availability of computer code

Data collection	Access to a comprehensive list of available corpora is requested
Data analysis	Data is analyzed with the R package brms (https://paul-buerkner.github.io/brms/). Acoustic properties were computed using OpenSMILE (https://audeering.github.io/opensmile/)

For manuscripts utilizing custom algorithms or software that are central to the research but not yet described in published literature, software must be made available to editors and reviewers. We strongly encourage code deposition in a community repository (e.g. GitHub). See the Nature Portfolio guidelines for submitting code & software for further information.

Data

Policy information about availability of data

All manuscripts must include a data availability statement. This statement should provide the following information, where applicable:

- Accession codes, unique identifiers, or web links for publicly available datasets
- A description of any restrictions on data availability
- For clinical datasets or third party data, please ensure that the statement adheres to our policy

The list of 200 requested corpora is available here: https://emotional-prosody.s3.amazonaws.com/corpora/index.html

Field-specific reporting

Please select the one below that is the best fit for your research. If you are not sure, read the appropriate sections before making your selection.

☐ Life sciences ☒ Behavioural & social sciences ☐ Ecological, evolutionary & environmental sciences

For a reference copy of the document with all sections, see nature.com/documents/nr-reporting-summary-flat.pdf

Behavioural & social sciences study design

All studies must disclose on these points even when the disclosure is negative.

Study description	A quantitative examination of the mapping between emotions and speech prosody.
Research sample	3,252 minutes of speech recordings, including 432 individuals (204 females, age not specified for all speakers) from 16 countries, speaking 2,963 different sentences.
Sampling strategy	In order to query all available corpora capturing emotional prosody, we combined three search strategies (see appendix for more details) with a final manual selection procedure. Each corpus included in the list needs to: (i) contain an annotation of the intended emotion by the speaker, and (ii) consist of recordings of sentences (i.e., not a syllable, non-verbal vocalization, a phoneme or a word).
Data collection	If the corpora were not openly available, we contacted the corresponding author of the corpus and signed a license agreement to use their corpus in this study.
Timing	All database queries were performed on April 1st, 2020.
Data exclusions	No data were excluded.
Non-participation	No participants dropped out.
Randomization	Participants were not allocated into experimental groups.

Reporting for specific materials, systems and methods

We require information from authors about some types of materials, experimental systems and methods used in many studies. Here, indicate whether each material, system or method listed is relevant to your study. If you are not sure if a list item applies to your research, read the appropriate section before selecting a response.

Materials & experimental systems

n/a	Involved in the study	
☒	☐	Antibodies
☒	☐	Eukaryotic cell lines
☒	☐	Palaeontology and archaeology
☒	☐	Animals and other organisms
☐	☒	Human research participants
☒	☐	Clinical data
☒	☐	Dual use research of concern

Methods

n/a	Involved in the study	
☒	☐	ChIP-seq
☒	☐	Flow cytometry
☒	☐	MRI-based neuroimaging

Human research participants

Policy information about studies involving human research participants

Population characteristics	See above.
Recruitment	Corpora included in the analysis had different recruitment strategies.
Ethics oversight	See publication of the corpus.

Note that full information on the approval of the study protocol must also be provided in the manuscript.
